# Intersectional inequalities in childhood maltreatment and adolescent emotional problems in England: a random-coefficient MAIHDA analysis

**DOI:** 10.1017/S2045796026100699

**Published:** 2026-06-04

**Authors:** Laura Havers, Mina Fazel, Melanie Smuk, Daisy Fancourt, Peter Fonagy, Paul McCrone, Gabriela Pavarini, Kamaldeep Bhui, Georgina M. Hosang, Sania Shakoor

**Affiliations:** 1 Centre for Psychiatry and Mental Health, Wolfson Institute of Population Health, Queen Mary, University of London, London, UK; 2 Department of Psychiatry, University of Oxford, Oxford, UK; 3 Centre for Genomics and Child Health, Blizard Institute, Queen Mary, University of London, London, UK; 4 Department of Behavioural Science and Health, University College London, London, UK; 5 Anna Freud National Centre for Children and Families, London, UK; 6 Research Department of Clinical, Educational and Health Psychology, University College London, London, UK; 7 Institute for Lifecourse Development, University of Greenwich, London, UK; 8 Department of Social Policy and Intervention, University of Oxford, Oxford, UK; 9 Department of Psychiatry, Nuffield Department of Primary Care Health Sciences, and Wadham College, University of Oxford, Oxford, UK; 10 Oxford Health and East London NHS Foundation Trusts, Oxford, UK; 11 World Psychiatric Association Collaborating Centre, Oxford, UK

**Keywords:** adolescence, inequalities, maltreatment, mental health, social factors

## Abstract

**Aims:**

Childhood maltreatment and adolescent mental health problems are unequally distributed, with the highest burdens among marginalised groups including females and those experiencing socioeconomic disadvantage. However, little is known about how the psychological consequences of maltreatment vary across intersecting social positions (e.g., socioeconomically disadvantaged females). Prior quantitative work has largely focused on average differences across a limited number of groups, obscuring non-additive intersectional patterning. Because social realities are structured by overlapping systems of privilege and oppression (e.g., relating to gender, socioeconomic position, ethnicity, age, and place), we leveraged recent methodological advances to address this gap. Accordingly, this study aimed to (i) map inequalities in adolescent emotional problems and the effects of maltreatment across intersectional positions; and (ii) describe the extent to which inequalities in emotional problems reflect additive and non-additive (intersectional) effects.

**Methods:**

Data were analysed from 19 590 students aged 11–16 years who participated in the OxWell 2023 Student Survey in England, United Kingdom. Within a random-coefficient Multilevel Analysis of Individual Heterogeneity and Discriminatory Accuracy (MAIHDA), individuals were nested in 180 intersectional strata defined by combinations of social positions relating to gender, ethnicity, household poverty, school year group, and school-level deprivation (also entered as additive main effects). Emotional problems (Revised Child Depression and Anxiety Scale; RCADS-11) were regressed on maltreatment exposure (Short Childhood Maltreatment Questionnaire) as the primary effect of interest. Stratum-specific predicted emotional problems and maltreatment effects were estimated, and between-stratum variance was partitioned into additive and residual non-additive components.

**Results:**

Maltreatment was associated with higher levels of emotional problems, with stratum-specific increases ranging from +3.20 to +6.14 scale points. Socioeconomically disadvantaged females and individuals who selected ‘other’ or ‘prefer not to say’ for gender showed the highest levels of emotional problems and among the strongest maltreatment effects. Between-stratum inequalities in emotional problems were largely accounted for by maltreatment exposure and the additive contributions of the included social positions. However, residual non-additive effects were also evident, particularly among individuals exposed to maltreatment, where 5.25% of between-stratum variance remained unaccounted for by additive effects (compared with 3.46% among those not exposed).

**Conclusions:**

In this large community sample of adolescents in England, the detrimental effects of maltreatment on emotional problems appear pervasive but not uniform across intersectional social positions. Applying an intersectional MAIHDA framework suggests that inequalities in adolescent emotional problems largely reflect additive social patterning, with additional non-additive contributions suggestive of intersectional dynamics that are more pronounced with maltreatment exposure. These findings motivate deeper investigation into the social-structural mechanisms that shape vulnerability and resilience in adolescence, and support the need for trauma-informed, equity-focused interventions and policy action to reduce unequal exposure to maltreatment and the contexts that amplify its harms.

## Introduction

Child maltreatment is a major social determinant of mental ill health (Carr *et al.*, 2020; Kirkbride *et al.*, 2024) and is alarmingly prevalent, with up to two-thirds of children and adolescents exposed (Stoltenborgh *et al.*, 2015; UNICEF, 2024). Maltreatment is defined as emotional and physical abuse and neglect within relationships of trust, responsibility and power (World Health Organization, 2022). This severe form of adversity disproportionately affects marginalised populations, particularly those experiencing socioeconomic disadvantage (Walsh *et al.*, 2019), racially and ethnically minoritised groups (Madigan *et al.*, 2023), females, and gender diverse individuals (Lipson *et al.*, 2019; Giano *et al.*, 2023). These inequities likely contribute to the unequal distribution of mental health problems among these populations (Kirkbride *et al.*, 2024). Yet despite substantial evidence on the social patterning of maltreatment and mental health problems (World Health Organization, 2008), relatively little is known about how intersecting forms of disadvantage structure the relation between maltreatment and adolescent mental health. Addressing this gap is critical for informing targeted, equity-focused interventions that reflect real-world conditions and diverse lived experiences (Hankivsky *et al.*, 2014).

Intersectionality theory provides a framework for understanding how individual characteristics (e.g., gender identity, race/ethnicity) serve as proxies for social positions shaped by interlocking systems of privilege and oppression (Crenshaw, 1990). Rather than operating in isolation, these systems intersect to situate individuals differently within hierarchies of power in ways that are not simply additive. For instance, the combined effect of being female and from an ethnoracially minoritised group may be greater, or less, than what would be expected based on the sum of each factor (King, 1988). Importantly, this does not diminish the structural weight of individual dimensions of inequality (e.g., persistent gender gaps) that warrant rigorous mechanistic investigation, even as intersectional analysis seeks to uncover deeper complexity (Patalay and Demkowicz, 2023). As a developmental period characterised by the emergence of social identity (Lerner and Galambos, 1998) and the onset of mental health problems (Caspi *et al.*, 2020), adolescence represents an important stage for intersectional analysis of maltreatment and its mental health consequences (Hosang *et al.*, 2023).

Evidence from this emerging line of inquiry suggests that adverse experiences, including maltreatment, and mental health problems are most common among young people at the intersectional societal margins (e.g., socioeconomically disadvantaged neurodivergent females) (Havers *et al.*, 2024a, 2024b). Intersectional patterns involving ethnicity appear more complex (Havers *et al.*, 2025), further emphasising a key principle of intersectionality theory that individuals can simultaneously occupy positions of both privilege and marginalisation (e.g., White females, Black males) (Bauer, 2014). Other findings indicate that ethnoracial inequalities may be amplified under particular conditions such as immigration (Slopen *et al.*, 2016), highlighting the need to situate intersectional research within broader historical, social, and political contexts.

Further, at the area level, adverse experiences and mental health problems are often most prevalent in urban and socioeconomically deprived regions (Weich *et al.*, 2006; Lewer *et al.*, 2020), which calls for investigation into how area-level and place-based characteristics that index inequities (e.g., crime, educational opportunities, access to services) interact with individual social positions (Camacho and Henderson, 2022). Such analyses are needed to understand how multiple forms of disadvantage and/or advantage converge to perpetuate inequalities, or mitigate their harm, in adolescent mental health outcomes (Diez Roux, 2001; Solar and Irwin, 2010).

Beyond shaping exposure to maltreatment and mental health outcomes, the *relation* between maltreatment and mental health may also vary across intersectional social positions. Examining these differences from an intersectional perspective not only helps identify the most vulnerable groups but also shifts the focus away from identity-based risk factors and toward the overlapping social-structural conditions that produce inequalities (Cole, 2009; Evans *et al.*, 2018). Recent findings from our group, based on a limited set of profiles, suggest possible intersectional effects involving gender, socioeconomic background, and neurodivergence in moderating the relation between youth adverse experiences and emotional problems (Havers *et al.*, 2024a, 2024b). These findings highlight the need for systematic investigation using enhanced modelling approaches to map how intersecting social-structural processes shape inequalities in adolescent mental health.

### Current study

Against this backdrop, it remains unclear the extent to which inequalities in adolescent emotional problems, and their relation to maltreatment, vary across intersectional social positions and whether any such variation reflects additive or non-additive (intersectional) processes. To address this, the current study applied Multilevel Analysis of Individual Heterogeneity and Discriminatory Accuracy (MAIHDA; Evans *et al.*, 2018). In this framework, individuals are nested within combinations of social dimensions of inequality (gender, ethnicity, socioeconomic position, age, and area-level characteristics, in this study), which are treated as higher-level units (strata) in a multilevel model. Unlike traditional fixed-effects regression approaches, MAIHDA readily accommodates high dimensionality and partitions between-stratum variation into additive and non-additive intersectional components to provide a descriptive starting point for understanding the social-structural conditions that drive inequalities (Evans and Erickson, 2019). Throughout, the term ‘effects’ is used to refer to conditional statistical estimates rather than causal effects, which is further commented on in the Discussion.

To our knowledge, this is the first study to apply MAIHDA to investigate the relation between maltreatment and adolescent mental health across intersectional social positions. The study aimed to: (1) map emotional problems among adolescents with and without maltreatment exposure across strata and estimate stratum-specific effects of maltreatment; and (2) describe the extent to which between-stratum variation in emotional problems is accounted for by (a) exposure to maltreatment, (b) the additive contributions of the included social positions and (c) residual effects beyond additivity, suggestive of intersectional dynamics.

## Methods

Minor deviations from the study preregistration (https://osf.io/8ryzw) are detailed in Supplementary Materials S1.

### Data source

The study uses data from the secondary school section of the 2023 OxWell Student Survey (hereafter OxWell) (https://oxwell.org/). OxWell is a repeated cross-sectional survey of young people in England, representative of those attending state schools in participating counties (Mansfield *et al.*, 2021). All mainstream state schools in participating counties were informed about the survey and invited through their local authorities, and all pupils within participating institutions were eligible to take part. The survey was administered (online) during school hours between February and March 2023 following procedures outlined in the pilot survey (Mansfield *et al.*, 2021). Eighty institutions across four main areas (Liverpool, Oxfordshire, Berkshire, Milton Keynes) participated. Because initial contact with schools was coordinated by local authorities, it was not possible to determine school-level participation rates for the 2023 survey. Estimates from the pilot survey within Oxfordshire suggest school- and pupil-level participation rates of approximately 10% and 85%, respectively (Mansfield *et al.*, 2021) (also see Discussion).

### Sample

Individuals in school Years 7–11 (ages 11–16) with complete data on gender, ethnicity, and household poverty were included (*n* = 19 682), as these variables defined the intersectional strata. School year and school-level deprivation data were available for all individuals. The analytic sample was drawn from a larger pool of 28 334 respondents (1% missing gender, 20% missing ethnicity, 14% missing household poverty). Supplementary Materials S2 provides a description of emotional problems and maltreatment among excluded individuals. After applying minimum stratum size thresholds (see description of intersectional strata), 19 590 individuals were retained.

### Ethics

Participants received study information and provided informed consent (aged 16) or assent (aged 11–15), with parents given the opportunity to opt their child out. No identifiable data were collected (Mansfield *et al.*, 2021). Ethical approval was granted by the University of Oxford Research Ethics Committee (reference: R62366/RE014).

### Measures

Emotional problems were assessed using the 11-item self-report Revised Child Depression and Anxiety Scale (RCADS-11) (Radez *et al.*, 2021). This scale asks about the frequency of anxiety (e.g., ‘I worry about what is going to happen’) and depressive (e.g., ‘I feel sad a lot’) symptoms, rated on a 4-point scale (‘Never’, ‘Sometimes’, ‘Often’, ‘Always’). Total scores were used, with participant-level mean imputation (Supplementary Materials S3).

Childhood maltreatment (hereafter maltreatment) was measured using the Short Child Maltreatment Questionnaire (Meinck *et al.*, 2016), covering current or past experiences of neglect, abuse, and witnessing domestic violence. The original item on specific forms of sexual abuse was omitted from OxWell for ethical and practical reasons. Participants responded whether each event had happened in their lifetime, in the past 12 months, never, or whether they would prefer not to say (Neelakantan *et al.*, 2025). A binary indicator of maltreatment was created to reflect any reported experience (Supplementary Materials S4).

Indicators of social position (detailed in Supplementary Materials S5; self-reported unless otherwise noted): *gender* (female, male, other, and prefer not to say [hereafter other/PNS]); *ethnicity* (White, Black, South Asian, Other Asian, Mixed, and Other); *household poverty* (binary indicator based on any reported experience using items from Wales’s Young People’s Survey on Child & Family Poverty (2019) and supplemented with one OxWell item); *school year* (Years 7–9; ages 11–14, and Years 10–11; ages 14–16); and *school-level deprivation* (indexed by the national-level Office for National Statistics Index of Multiple Deprivation [IMD] deciles, recoded into three categories representing the three most deprived deciles, the middle four deciles, and the three least deprived deciles) https://www.gov.uk/government/statistics/english-indices-of-deprivation-2015). IMD served as both a substantive indicator and a proxy for school-level variation.

Intersectional strata were defined by combinations of the above social positions, yielding 216 strata. To minimise risk of identifiability, school-level identifiers were not included, and only strata with five or more individuals (*n* = 180) were retained (Supplementary Materials S6). The distribution of individuals across strata is consistent with recommendations (Evans *et al.*, 2024), with 86% of strata containing at least 10 individuals and 59% at least 30 (Table 1).

**Table 1.** Characteristics of included intersectional strata (*n* = 180) Table 1 long description.

Number of individuals in stratum	Number of strata	% of total strata
5–9	26	14.44%
10–29	48	26.67%
30–99	66	36.67%
100–499	31	17.22%
500–999	6	3.33%
1 000–1 310	3	1.67%

### Statistical analyses

The main analyses used a random-coefficient extension of MAIHDA (Evans *et al.*, 2023, 2024) implemented with maximum likelihood estimation. Individuals (level 1) were nested within intersectional strata (level 2). Five models were specified sequentially. A null model (Model 1) partitioned variance in emotional problems into within- and between-stratum components. Model 2 included maltreatment as a fixed effect, assuming its effect on emotional problems was constant across strata. Model 3 extended this by allowing maltreatment to vary randomly across strata. Model 4 returned to the fixed maltreatment effect specification and added the indicators of social position (gender, ethnicity, household poverty, school year, and school-level deprivation) as additive fixed effects. Model 5 extended Model 4 by allowing maltreatment to vary randomly across strata while retaining the additive fixed effects of the included social positions.

For the first study aim, stratum-specific levels of emotional problems and stratum-specific maltreatment effects were estimated using empirical Bayes predictions from Model 3, with approximate confidence intervals derived from asymptotic standard errors. These estimates combined the average (fixed) intercept and maltreatment effect with stratum-level (random) deviations to generate predicted levels of emotional problems with and without maltreatment exposure for each stratum.

For the second aim, variance-decomposition metrics were derived from sequential models to quantify sources of between-stratum variance in emotional problems (Evans *et al.*, 2023, 2024). The Variance Partition Coefficient (VPC) indexed the proportion of total variance attributable to between-stratum differences. The Proportional Change in Variance (PCV) quantified the proportion of between-stratum variance accounted for by adding the model-specific main effects. In Models 4 and 5, the residual PCV (1 − PCV) reflected the proportion of between-stratum variance unaccounted for by additive effects, interpreted as intersectional effects. Detailed descriptions of VPC and PCV values for each model are presented in Supplementary Materials S7. R code for the MAIHDA models, adapted from Evans and colleagues’ foundational code (2024) and random-coefficient formulations (2023) is stored at https://osf.io/8ryzw/files/osfstorage.

Multilevel multiple imputation, accounting for the clustering of individuals within intersectional strata, was used to accommodate missing data for maltreatment (29%) and emotional problems (16%; and both: 14%) under the assumption of missingness at random conditional on emotional problems, maltreatment, gender, ethnicity, poverty, year group, and IMD data. From an initial run of 30 imputations, three imputations returned zero stratum-level variance estimates and three had convergence issues. These runs were excluded, and a random sample of 20 imputations from the remaining valid set was retained, with a burn-in of 15 000 iterations and 2 000 iterations between imputations (R script stored at https://osf.io/8ryzw/files/osfstorage). Final analyses were conducted on pooled data and estimates adjusted using Rubin’s rules (Rubin, 1976). All analyses were carried out in R version 4.4 (R Core Team, 2024), using *lme4* for multilevel models (Bates *et al.*, 2015) and *jomo* for multiple imputation (Quartagno and Carpenter, 2023).

## Results

### Description

Sample characteristics and descriptive statistics are presented in Table 2. More than one-third of the sample (*n* = 6 829; 34.86%) identified as non-White, and 23.33% experienced household poverty (*n* = 4 570). One quarter reported exposure to maltreatment (*n* = 5 043; 25.7%). Stratum-specific levels are provided in Supplementary Table 1.

**Table 2.** Sample characteristics and emotional problems by maltreatment exposure Table 2 long description.

	**Total** (*n* *=* 19 590)		**Maltreated** (*n* = 5 043)		**Not maltreated** (*n* = 8 892)		**Missing maltreatment data** (*n* = 5 655)	
	*n* (%)	Emotional problems mean (SD)	*n* (%)	Emotional problems mean (SD)	*n* (%)	Emotional problems mean (SD)	*n* (%)	Emotional problems mean (SD)
**Individuals with emotional problems data**	16 499 (84.22%)	10.54 (8.22)	4 955 (98.26%)	14.46 (8.58)	8 716 (98.02%)	8.37 (7.13)	2 828 (50.01%)	10.34 (8.19)
**Gender**								
Male	8 636 (44.08%)	7.49 (6.79)	1 870 (37.08%)	10.45 (7.63)	3 893 (44.79%)	6.09 (5.85)	2 783 (49.21%)	7.55 (6.84)
Female	10 185 (51.99%)	12.51 (8.30)	2 888 (57.27%)	16.48 (8.10)	4 626 (52.02%)	10.00 (7.40)	2 671 (47.23%)	12.57 (8.32)
Other and prefer not to say	769 (3.93%)	16.85 (9.47)	285 (5.65%)	20.07 (8.81)	283 (3.18%)	13.72 (8.82)	201 (3.55%)	16.25 (10.15)
**Ethnicity**								
White	12 761 (65.14%)	10.83 (8.41)	3 209 (63.63%)	14.90 (8.74)	5 928 (66.67%)	8.61 (7.35)	3 624 (64.08%)	10.87 (8.35)
Mixed Ethnicity	1 354 (6.91%)	11.38 (8.14)	448 (8.88%)	14.56 (8.31)	508 (5.71%)	8.89 (6.90)	398 (7.04%)	10.49 (8.30)
South Asian	2 309 (11.79%)	9.50 (7.85)	566 (11.22%)	13.89 (8.53)	1 144 (12.87%)	7.55 (6.59)	599 (10.59%)	8.67 (7.64)
Other Asian	1 077 (5.50%)	10.02 (7.37)	308 (6.11%)	13.01 (7.55)	475 (5.34%)	8.11 (6.42)	294 (5.20%)	10.02 (7.83)
Black	1 145 (5.84%)	9.58 (7.62)	316 (6.27%)	13.13 (7.89)	423 (4.76%)	7.23 (6.50)	406 (7.18%)	8.88 (7.27)
Other Ethnicity groups	944 (4.82%)	9.64 (7.87)	196 (3.89%)	13.08 (8.59)	414 (4.66%)	8.02 (6.85)	334 (5.91%)	9.59 (8.11)
**Household poverty**								
No	15 020 (76.67%)	9.12 (7.52)	3 247 (64.39%)	12.73 (8.10)	7 546 (84.86%)	7.67 (6.77)	4 227 (74.75%)	8.76 (7.31)
Yes	4 570 (23.33%)	15.26 (8.68)	1 796 (35.61%)	17.59 (8.54)	1 346 (15.14%)	12.33 (7.83)	1 428 (25.25%)	14.88 (8.86)
**School year group** (average age range)								
Years 7–9 (11–14-years)	13 274 (67.76%)	10.12 (8.10)	3 235 (64.15%)	14.21 (8.68)	6 250 (70.29%)	7.98 (6.92)	3 789 (67.00%)	10.13 (7.99)
Years 10–11 (14–16 years)	6 316 (32.24%)	11.44 (8.40)	1 808 (35.85%)	14.91 (8.38)	2 642 (29.71%)	9.28 (7.53)	1 866 (33.00%)	10.81 (8.59)
**School-level deprivation** (IMD)								
Least deprived	7 885 (40.25%)	10.66 (8.23)	2 076 (41.17%)	14.50 (8.63)	3 443 (38.72%)	8.45 (7.05)	2 366 (41.84%)	10.34 (8.25)
Mid-deprived	5 178 (26.43%)	10.02 (8.06)	1 378 (27.33%)	13.82 (8.47)	2 455 (27.61%)	7.93 (6.98)	1 345 (23.78%)	9.85 (8.09)
Most deprived	6 527 (33.32%)	10.82 (8.32)	1 589 (31.51%)	14.98 (8.57)	2 994 (33.67%)	8.64 (7.34)	1 944 (34.38%)	10.69 (8.18)

*Notes:* Emotional problems indexed by the Revised Child Depression and Anxiety Scale (RCADS-11). Maltreatment indexed by the Short Childhood Maltreatment Questionnaire (SCMQ). IMD; Index of Multiple Deprivation.

The mean score for emotional problems was 10.54 (SD = 8.22). Scores were higher in females (*M* = 12.51, SD = 8.30) and other/PNS individuals (*M* = 16.85, SD = 9.47), and lower in males (*M* = 7.49, SD = 6.79). Those experiencing household poverty reported higher scores (*M* = 15.26, SD = 8.68) compared to those not in poverty (*M* = 9.12, SD = 7.52). Mean scores were broadly similar across ethnic groups, year groups, and levels of school-level deprivation. Disparities by gender and poverty were amplified among individuals exposed to maltreatment.

### Aim 1: predicted stratum-specific emotional problems and maltreatment effects

Table 3 shows the strata with the highest and lowest predicted emotional problems (all strata are reported in Supplementary Tables 2–3). Among those exposed to maltreatment, the highest emotional problem scores (22.49–24.48) were observed in strata of other/PNS individuals experiencing household poverty, mostly from schools in the most deprived areas (excluding one). The lowest scores (7.57–8.06) were observed among strata of non-White males without household poverty.

**Table 3.** Highest and lowest predicted emotional problems for young people exposed and not exposed to maltreatment, and largest and smallest differences in predicted emotional problems by maltreatment Table 3 long description.

**Highest predicted emotional problems**				**Lowest predicted emotional problems**			
**Individuals exposed to maltreatment**		**Individuals not exposed to maltreatment**		**Individuals exposed to maltreatment**		**Individuals not exposed to maltreatment**	
**Stratum**	**Predicted emotional problems**	**Stratum**	**Predicted emotional problems**	**Stratum**	**Predicted emotional problems**	**Stratum**	**Predicted emotional problems**
OtherPNS-White-Poverty-Y10-11-LeastDeprived	24.48 [22.82, 26.14]	OtherPNS-White-Poverty-Y10-11-LeastDeprived	18.58 [16.77, 20.40]	Male-Black-NoPoverty-Y7-9-MidDeprived	7.57 [6.39, 8.76]	Male-Black-NoPoverty-Y7-9-MidDeprived	3.70 [2.50, 5.09]
OtherPNS-White-Poverty-Y10-11-MostDeprived	24.19 [22.92, 25.47]	OtherPNS-White-Poverty-Y10-11-MostDeprived	18.25 [17.01, 19.50]	Male-Black-NoPoverty-Y7-9-LeastDeprived	7.72 [5.93, 9.51]	Male-Black-NoPoverty-Y10-11-MostDeprived	4.01 [2.57, 5.44]
OtherPNS-Mixed-Poverty-Y7-9-MostDeprived	23.62 [20.86, 26.83]	OtherPNS-Mixed-Poverty-Y7-9-MostDeprived	17.79 [15.23, 20.35]	Male-Black-NoPoverty-Y10-11-MostDeprived	7.85 [6.29, 9.40]	Male-S.Asian-NoPoverty-Y7-9-MostDeprived	4.09 [3.39, 4.79]
OtherPNS-White-Poverty-Y7-9-MostDeprived	22.63 [21.82, 23.44]	OtherPNS-White-Poverty-Y7-9-MostDeprived	17.18 [16.25, 18.12]	Male-S.Asian-NoPoverty-Y7-9-MostDeprived	8.05 [6.85, 9.25]	Male-Black-NoPoverty-Y7-9-LeastDeprived	4.24 [2.94, 5.55]
OtherPNS-Black-Poverty-Y7-9-MostDeprived	22.49 [21.98, 23.01]	OtherPNS-Black-Poverty-Y7-9-MostDeprived	16.78 [15.97, 17.59]	Male-O.Asian-NoPoverty-Y10-11-MostDeprived	8.06 [6.01, 10.12]	Male-O.Asian-NoPoverty-Y7-9-LeastDeprived	4.30 [3.03, 5.57]
**Maltreatment effects (*β*_1j_) (predicted differences)**							
**Largest predicted differences**				**Smallest predicted differences**			
**Stratum**	**Individuals exposed to maltreatment**	**Individuals not exposed to maltreatment**	**Difference**	**Stratum**	**Individuals exposed to maltreatment**	**Individuals not exposed to maltreatment**	**Difference**
Female-White-NoPoverty-Y7-9-LeastDeprived	15.64 [15.04, 16.24]	9.50 [9.26, 9.74]	6.14 [5.48, 6.80]	Male-Black-NoPoverty-Y7-9-MostDeprived	8.19 [7.35, 9.04]	4.99 [4.03, 5.96]	3.20 [2.60, 3.80]
Female-S.Asian-Poverty-Y7-9-MostDeprived	20.88 [20.00, 21.77]	14.81 [13.97, 15.65]	6.07 [5.60, 6.55]	Male-White-NoPoverty-Y10-11-LeastDeprived	9.50 [9.03, 9.97]	6.09 [5.65, 6.52]	3.42 [2.91, 3.92]
OtherPNS-White-Poverty-Y10-11-MostDeprived	24.19 [22.92, 25.47]	18.25 [17.01, 19.50]	5.94 [4.97, 6.91]	Male-White-NoPoverty-Y7-9-LeastDeprived	8.95 [8.47, 9.42]	5.49 [5.25, 5.73]	3.45 [2.82, 4.08]
OtherPNS-White-Poverty-Y10-11-LeastDeprived	24.48 [22.82, 26.14]	18.58 [16.77, 20.40]	5.89 [4.95, 6.84]	Male-Black-NoPoverty-Y7-9-LeastDeprived	7.72 [5.93, 9.51]	4.24 [2.94, 5.55]	3.47 [2.63, 4.32]
OtherPNS-Mixed-Poverty-Y7-9-MostDeprived	23.62 [20.86, 26.38]	17.79 [15.23, 20.35]	5.83 [5.00, 6.66]	Male-White-Poverty-Y7-9-MostDeprived	13.65 [12.69, 14.61]	10.07 [9.26, 10.88]	3.58 [2.57, 4.59]

*Notes:* Emotional problems indexed by the Revised Child Depression and Anxiety Scale (RCADS-11). 95% approximate CI in square brackets. Stratum label order: gender, ethnicity, household poverty, school year group, school-level deprivation. OtherPNS = other and prefer not to say; S.Asian = South Asian; O.Asian = Other Asian. Estimates derived from Model 3. The effect of maltreatment (*β*_1j_) is equivalent to the predicted difference in emotional problems between individuals exposed and not exposed to maltreatment. Predicted values for all strata are reported in Supplementary Tables 2–4.

Among those not exposed to maltreatment, the ranking of the highest strata (16.78–18.58) was preserved, consisting of other/PNS individuals experiencing poverty, mostly from schools in the most deprived areas. The strata with the lowest scores (3.70–4.30) were broadly the same as for individuals exposed to maltreatment, with minor reshuffling in order.

Maltreatment appeared detrimental across all strata, though the magnitude of the effect (predicted differences in emotional problems) varied (Supplementary Table 4). The strongest stratum-specific effects (*β*_1j_ = 5.83–6.14) occurred in strata of females or other/PNS individuals, predominantly among those experiencing poverty (with one exception). The weakest effects (*β*_1j_ = 3.20–3.58) were observed among strata of males not experiencing poverty. No consistent patterns were found for ethnicity, year group, or school-level deprivation.

**Figure 1.**
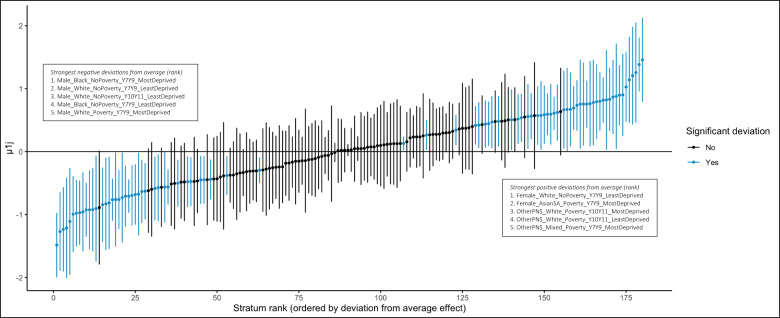
Stratum-level deviations from the average effect of maltreatment on emotional problems in each intersectional stratum. Figure 1 long description.

*Notes*. Emotional problems indexed by the Revised Child Depression and Anxiety Scale (RCADS-11). Stratum-level deviations (*μ*_1j_) are estimated in Model 3, where the average effect of maltreatment across strata (*β*_1_) is 4.69 [95% CI: 4.30, 5.07]. Points above the horizontal line (*μ*_1j_ = 0) reflect larger stratum-level effects of maltreatment than the average effect, and deviations below the line reflect smaller stratum-level effects of maltreatment than the average. Vertical lines represent 95% approximate CI. Blue points and CI indicate non-overlap with *μ*_1j_ = 0. Callout labels: The strongest negative deviations correspond to the points running from left to right in the figure, and the strongest positive deviations correspond to the points running from right to left in the figure. Numerical values are reported in Supplementary Table 5

Figure 1 illustrates stratum-specific deviations (*μ*_1j_) from the average effect of maltreatment (*β*_1_ = 4.69 [95% CI 4.30, 5.07]), with numerical values in Supplementary Table 5. Ethnicity, poverty, year group and school-level deprivation were distributed across both positive and negative deviations. However, most strata with significant positive deviations (indicating greater effects than the average) included females or other/PNS individuals, whereas most strata with significant negative deviations included males. At the extremes, the largest positive deviation (*μ*_1j_ = 1.51 [95% CI 0.97, 2.06]) was observed in younger White females without poverty in schools from the least deprived areas, while the largest negative deviation (*μ*_1j_ = −1.40 [95% CI −2.07, −0.73]) was observed in younger Black males without poverty in schools from the most deprived areas.

### Aim 2: partitioning variance in emotional problems

Results of the MAIHDA models are presented in Table 4. In Model 1, the VPC indicated that 26.98% of the total variance in emotional problems was attributable to differences between intersectional strata. In Model 2, emotional problems were 4.63 units higher on average among individuals exposed to maltreatment compared to those not exposed. With maltreatment included as a fixed effect, 23.43% of the variance in emotional problems remained attributable to strata. Between-stratum variance decreased from 19.59 in Model 1 to 14.86 in Model 2, corresponding to a 24.16% PCV. This indicates that maltreatment accounted for roughly one quarter of the between-stratum variance in emotional problems, though substantial variation remained unaccounted for.

**Table 4.** Emotional problems in young people exposed and not exposed to maltreatment: intersectional MAIHDA models Table 4 long description.

	**Model 1**	**Model 2**	**Model 3**	**Model 4**	**Model 5**
	Null model	Maltreatment fixed effect	Maltreatment random coefficient	Maltreatment fixed effect + additive fixed effects	Maltreatment random coefficient + additive fixed effects
**Intercept**	12.19 [11.50, 12.87]	10.02 [9.40, 10.64]	9.95 [9.35, 10.54]	5.31 [4.82, 5.80]	5.33 [4.84, 5.81]
**Maltreatment** (SCMQ; ref: no maltreatment) Maltreatment		4.63 [4.35, 4.92]	4.69 [4.30, 5.07]	4.58 [4.29, 4.86]	4.46 [4.07, 4.86]
**Gender** (ref: male)					
Female				4.57 [4.19, 4.96]	4.57 [4.18, 4.96]
Other and prefer not to say				7.66 [6.96, 8.36]	7.68 [6.97, 8.38]
**Ethnicity** (ref: White)					
Mixed Ethnicity				−0.46 [−1.04, 0.11]	−0.43 [−1.00, 0.14]
South Asian				−1.17 [−1.70, −0.64]	−1.19 [−1.70, −0.67]
Other Asian				−0.94 [−1.56, −0.32]	−0.93 [−1.55, −0.31]
Black				−1.83 [−2.46, −1.20]	−1.81 [−2.42, −1.19]
Other ethnic groups				−1.32 [−1.98, −0.65]	−1.33 [−1.98, −0.67]
**Household poverty** (ref: no)					
Yes				4.56 [4.17, 4.94]	4.58 [4.20, 4.97]
**School year group** (ref: years 7–9)					
Years 10–11				1.00 [0.63, 1.37]	1.03 [0.67, 1.39]
**School-level deprivation** (IMD; ref: least deprived)					
Mid-deprived				−0.30 [−0.73, 0.13]	−0.29 [−0.72, 0.13]
Most deprived				−0.15 [−0.58, 0.28]	−0.15 [−0.56, 0.27]
**Random effects**					
Intersectional strata	19.59	14.86	13.26	0.38	0.46
Maltreatment slope			0.98		1.11
Covariance			1.77		−0.32
Individual (residual)	53.00	48.53	48.32	48.52	48.35
**Summary coefficients**					
VPC (%)	26.98	23.43	21.53	0.84	0.94
VPC (%): maltreatment			26.90		1.89
Between-stratum variance: no maltreatment			13.26		0.46
Between-stratum variance: maltreatment			17.78		0.93
PCV (%)		24.16		97.89^a^	
PCV (%): no maltreatment					96.54^b^
PCV (%): maltreatment					94.75^b^

*Notes*: Emotional problems indexed by the Revised Child Depression and Anxiety Scale (RCADS-11). SCMQ, Short Childhood Maltreatment Questionnaire; IMD, Index of Multiple Deprivation; VPC, Variance Partition Coefficient; PCV, proportional change in variance. For Model 5, PCV values are reported separately for individuals exposed to maltreatment and those not exposed to maltreatment. 95% CI in square brackets.

a PCV values reflect the proportional change in variance from Model 2.

b PCV values reflect the proportional change in variance from Model 3.

**Figure 2.**
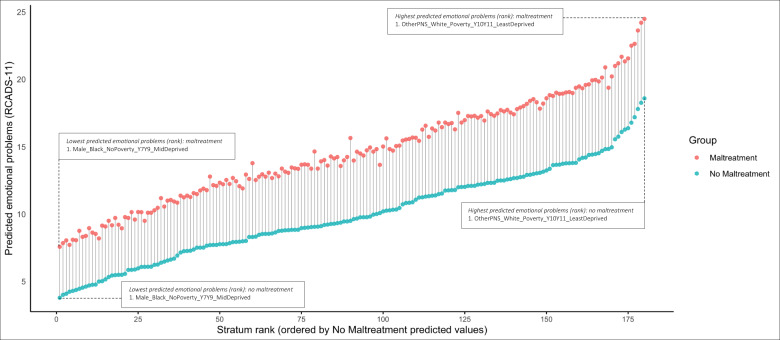
Predicted emotional problems for young people exposed and not exposed to maltreatment in each intersectional stratum. Figure 2 long description.

*Notes*. Emotional problems indexed by the Revised Child Depression and Anxiety Scale (RCADS-11). Predicted values estimated in Model 3. Numerical values are reported in Supplementary Tables 2 (no maltreatment) and 3 (maltreatment)

In Model 3, the effect of maltreatment on emotional problems was allowed to vary across strata. These stratum-specific differences (discussed above; Table 3) are presented visually in Fig. 2, further illustrating the between-stratum variance reflected in the VPCs. Among individuals exposed to maltreatment, 26.90% of the variance in emotional problems was attributable to between-stratum differences, compared to 21.53% for those not exposed, indicating greater between-stratum differences in emotional problems among individuals exposed to maltreatment.

In Models 4 and 5, the fixed effects of the social position indicators aligned with the descriptive observations (Table 2). Emotional problems were higher among females and other/PNS individuals compared with males, and among those experiencing poverty compared to those without. Higher scores were also observed among older adolescents (school years 10–11 vs. 7–9), and among White adolescents compared with South Asian, Other Asian, Black, and Other ethnicity groups.

In Model 5, 1.89% of the variance in emotional problems among maltreated individuals was attributable to between-stratum differences, compared with 0.94% among those not exposed. Of the between-stratum variance among maltreated individuals, 94.75% was explained by additive effects, with the remaining 5.25% reflecting residual non-additive effects. This proportion was higher than among non-maltreated individuals, where 3.46% of the between-stratum variance reflected non-additive effects (PCV = 96.54%). These results indicate that residual effects, suggestive of intersectional dynamics, remain after accounting for the additive effects of the included social positions, and that they constitute a larger share of between-stratum variance among individuals exposed to maltreatment than among those not exposed.

## Discussion

This study applied a quantitative intersectional framework to examine inequalities in the relation between childhood maltreatment and adolescent emotional problems. In this large sample of adolescents in England, the detrimental effects of maltreatment appeared pervasive but varied across intersectional strata. Socioeconomically disadvantaged females and other/PNS individuals carried the greatest mental health burden, with additional patterns of vulnerability and resilience observed. While most between-stratum differences in emotional problems were accounted for by the additive effects of the included social positions, evidence of non-additive effects suggests that inequalities may also at least partly depend on how social positions combine within broader structural conditions, particularly in the context of maltreatment. These findings underscore the importance of considering the complex dynamics of systems of oppression and power when addressing adolescents’ vulnerability and resilience to emotional problems.

Consistent with previous research (Carr *et al.*, 2020), maltreatment was associated with higher levels of emotional problems across all strata. Our findings extend this evidence by showing that both the absolute burden of emotional problems and the relative impact of maltreatment followed gradients consistent with multiple marginalisation (Evans *et al.*, 2013). Other/PNS adolescents experiencing household poverty reported the highest levels of emotional problems, regardless of maltreatment exposure, and some of the strongest maltreatment effects. Females experiencing household poverty were similarly among the most affected. The current findings illustrate how socioeconomic disadvantage and gender inequality may compound to intensify burden (Kirkbride *et al.*, 2024), and further suggest that area-level deprivation may further amplify risk among marginalised groups, warranting further investigation (Latham *et al.*, 2025). They also highlight the additional structural and psychosocial stressors faced by other/PNS individuals, where stigma, discrimination, and exclusion intersect with other inequalities to heighten vulnerability (Meyer, 2003; Hatzenbuehler, 2009). By contrast, non-White males not experiencing poverty consistently reported the lowest emotional problems and smallest maltreatment effects, suggesting that the relative social advantages associated with male gender and access to socioeconomic resources *may* help buffer against adversity even where ethnoracial minoritisation is present (Ungar, 2013). We return to the point of simultaneous privilege and oppression below.

Notably, maltreatment appeared to exacerbate emotional problems most strongly among multiply marginalised adolescents, in contrast to our prior work where broader adversity had greater detrimental effects among relatively more advantaged groups (Havers *et al.*, 2024a, 2024b). These differing patterns may indicate that maltreatment, as a specific and severe form of adversity (Norman *et al.*, 2012), is associated with different outcomes than cumulative exposures including bullying victimisation and parental separation. This interpretation is consistent with evidence that different adversities cluster and display distinct social patterning (Lacey *et al.*, 2022). Together, these findings indicate that maltreatment may be particularly damaging for socioeconomically disadvantaged female and other/PNS adolescents, extending recent evidence of heightened vulnerability among gender diverse youth (Madzoska *et al.*, 2024). Of note, use of the RCADS-11 may also have improved sensitivity to variation across social positions, especially among gender minoritised and socioeconomically disadvantaged groups, who report elevated emotional problems regardless of maltreatment exposure (Lipson *et al.*, 2019; Kern *et al.*, 2020).

Stratum-specific deviations showed that vulnerability and resilience to maltreatment relative to the average effect did not map neatly onto absolute levels of disadvantage. For example, younger White females without household poverty in the least deprived schools experienced larger-than-average effects of maltreatment, whereas younger Black males without poverty in more deprived schools experienced smaller-than-average effects. Although seemingly counterintuitive, these findings can be understood from an intersectional theoretical perspective, which emphasises that individuals may simultaneously occupy privileged and marginalised positions and that their interaction does not map directly onto expected patterns of risk or protection (Crenshaw, 1990). As Evans *et al.* (2018) note, intersectional experiences are not additive, and neither the number nor the type of social positions linearly predicts vulnerability. MAIHDA makes this complexity (descriptively) visible, highlighting that some groups with relatively low absolute burden (e.g., White females without household poverty) may experience disproportionately high maltreatment effects, while others with consistently high burden (e.g., other/PNS adolescents experiencing poverty) report elevated emotional problems regardless of maltreatment exposure. Together, these findings emphasise the need to move beyond additive models to understand how unique configurations of social positions shape vulnerability and resilience (Bauer, 2014; Evans *et al.*, 2018). This can challenge assumptions of uniform risk and contribute to theoretical models of how maltreatment affects adolescent mental health across diverse social contexts (Shimmin *et al.*, 2017).

Beyond mapping disparities between strata, a key strength of MAIHDA is its capacity to shed light on the statistical structuring of observed differences through a variance-decomposition approach. While this does not in itself test causal explanations for the mechanisms underlying these disparities, it provides a descriptive account of how such differences are structured in terms of additive and residual non-additive (intersectional) effects. In this study, a substantial share of variability in emotional problems was attributable to between-stratum differences. Approximately one quarter of this variance was accounted for by differential exposure to maltreatment, consistent with evidence that adversity is strongly socially patterned (Walsh *et al.*, 2019; Kirkbride *et al.*, 2024). However, most between-stratum variance remained unaccounted for, pointing to additional social and structural processes underlying the observed disparities. Stratum comparisons by maltreatment exposure further suggested that inequalities were greater among those exposed (26.9%) than those not exposed (21.5%), implying that maltreatment may amplify intersectional inequalities. At the same time, the considerable within-stratum variation that was observed indicates that adolescents with similar social positions may still experience markedly different levels of emotional problems (Evans *et al.*, 2023). This reinforces calls for more nuanced approaches to intervention and policy that address group-level inequalities while recognising within-group heterogeneity (Bryan *et al.*, 2021; National Scientific Council on the Developing Child, 2024). MAIHDA is well-suited to provide a starting point for such investigation by simultaneously estimating stratum-level inequalities, and individual-level variability (Evans *et al.*, 2023).

Most between-stratum differences in emotional problems were attributable to the additive effects of the included social positions reflecting gender, ethnicity, household poverty, year group, and school-level deprivation. This is important, as it highlights that single-axis dimensions, such as gender, remain central drivers of inequality (Patalay and Demkowicz, 2023) even as intersectional analyses reveal more nuanced dynamics. Notwithstanding, a non-trivial proportion of variation was attributable to the intersections of social positions. These intersectional dynamics (captured as residual effects) were more pronounced among adolescents exposed to maltreatment (5.25%) than among their non-maltreated peers (3.46%). This pattern suggests that the ways in which social positions and contexts combine beyond their additive contributions may generate unique constellations of vulnerability and/or resilience, particularly in the presence of maltreatment. Taken together, the findings support the value of trauma-informed, intersectional frameworks that recognise adverse experiences as inseparable from broader interlocking systems of inequality, with implications for designing targeted interventions (Shimmin *et al.*, 2017).

From an applied perspective, the results highlight several preliminary findings with potential relevance for practice and policy. At the school level, the concentration of the highest levels of emotional problems among socioeconomically disadvantaged adolescents reporting ‘other’ (than female/male) or ‘prefer not to say’ gender identities, points to the need for inclusive mental health support, anti-discrimination initiatives, and resources to support students facing both gender-based and socioeconomic social disadvantage, particularly in the most deprived schools. For child and adolescent mental health and child protection services, and related provision, evidence that the effects of maltreatment are strongest among female and other/PNS adolescents facing socioeconomic disadvantage, and that maltreatment appears to amplify mental health inequalities across strata, highlights the value of targeted outreach and tailored support for adolescents experiencing multiple forms of marginalisation alongside exposure to adverse experiences including maltreatment. At a policy level, the findings suggest that area-level inequities may compound social-structural disadvantage that is experienced at the individual-level within specific intersectional contexts, which may be particularly salient among socioeconomically disadvantaged other/PNS adolescents. This highlights the importance of integrating child protection and mental health strategies with wider social policies that address structural inequalities. More generally, the most effective responses to the unequal mental health consequences of maltreatment may be those that address both single-axis and intersectional systems of disadvantage, while remaining sensitive to variability within, as well as between, socially patterned groups (Shimmin *et al.*, 2017; Baldwin *et al.*, 2023; National Scientific Council on the Developing Child, 2024).

### Strengths and limitations

To our knowledge, this is among the first studies to examine the relation between maltreatment and adolescent mental health using an explicitly intersectional approach. Alongside the advantages of MAIHDA over traditional fixed-effects methods, this study benefits from a large sample of UK adolescents, detailed measures of maltreatment and emotional problems, and the inclusion of multiple indicators of intersectional positionality.

Several limitations should also be noted. Although MAIHDA can accommodate small strata sizes via shrinkage, it was still necessary to collapse some social position categories due to very small counts once other indicators were combined. For example, individuals of Black African and Black Caribbean heritage were grouped together, as were those with any mixed ethnic background, and those with ethnic backgrounds other than the other five categories used in this study. This aggregation may obscure meaningful within-group differences shaped by distinct historical, cultural, and structural factors (Aspinall, 2007; Ford and Harawa, 2010). A similar limitation applies to the aggregation of participants selecting ‘other’ or ‘prefer not to say’ for gender, which may mask important heterogeneity (Soneson *et al.*, 2024).

In addition, confidence intervals were derived from asymptotic standard errors, which may not fully capture uncertainty around the predicted values. Alternative approaches using Markov chain Monte Carlo estimation would provide a more comprehensive representation of uncertainty. Our substantive conclusions are unlikely to differ, however, as point estimates are generally consistent across estimation methods (Evans *et al.*, 2024).

Although excluded individuals had similar mean levels of emotional problems and maltreatment to those in the analytic sample (Supplementary Materials S2), the exclusion of individuals with missing social position data (primarily for ethnicity and household poverty) may have nonetheless resulted in a conservative descriptive picture of inequality, given that non-response for these variables is more likely among marginalised groups (Ahlmark *et al.,* 2015). Future work should consider the implications of, and where appropriate implement, an extended multiple imputation model to retain a greater number of marginalised individuals and reduce potential bias arising from complete-case analysis based on the included social position indicators.

Further, while the inclusion of multiple indicators of intersectional positionality builds on our prior work (Havers *et al.*, 2024a, 2024b, 2025), the findings remain limited to the social positions assessed. Residual stratum-level variation may capture not only non-additive (intersectional) patterning but also differences arising from unmeasured or unobserved dimensions of social position (Evans *et al.*, 2018). This may be particularly relevant for sexual orientation, for instance, given recent evidence of its importance in mental health inequalities (Moreno-Agostino *et al.*, 2024).

In addition, the results reflect a cross-sectional snapshot of UK secondary-school pupils enrolled in participating schools in 2023 and present on the day of survey administration. Although the individual-level opt-out design reduces selection bias relative to opt-in models, generalisability remains limited to pupils present on the survey day within participating institutions (Mansfield *et al.*, 2021). Some individuals may further have withheld responses to sensitive questions despite the survey’s anonymity (Soneson *et al.*, 2025).

While the temporal ordering of past maltreatment and current emotional problems supports a causal interpretation, given synthesised evidence indicating only partial causality (Baldwin *et al.*, 2023), future work should explicitly test causal models that account for shared upstream determinants and potential confounding (Kirkbride *et al.*, 2024). Relatedly, recall bias and reinterpretation of events cannot be ruled out, although the use of specific and objectively worded items is likely to reduce such risks (Gilbert *et al.*, 2009).

## Conclusion

The current findings indicate that the relation between maltreatment and adolescent emotional problems is not uniform but varies across intersecting social positions, with the highest burdens observed among socioeconomically disadvantaged females and individuals who selected ‘other’ or ‘prefer not to say’ for gender. By demonstrating both additive and non-additive sources of inequality, this study highlights the complexities of understanding vulnerability and resilience and provides a platform for further investigation into the social-structural mechanisms that underlie maltreatment and its unequal psychological consequences.

## Supporting information

10.1017/S2045796026100699.sm001 Havers et al. supplementary material Havers et al. supplementary material

## Data Availability

The data that support the findings are available upon reasonable request. Researchers may access the data by applying through the BrainWaves Data Portal (https://brainwaveshub.org/for-research/), where applications are reviewed to ensure appropriate use. Further details, including the full list of questions, study protocol, and other supporting materials, are available via the OxWell project’s Open Science Framework page: https://osf.io/sekhr/. R code for the multiple imputation model and MAIHDA models [adapted from Evans and colleagues’ foundational code (2024) and random-coefficient formulations (2023)], is available at https://osf.io/8ryzw/files/osfstorage.

## Supplementary material

The supplementary material for this article can be found at https://doi.org/10.1017/S2045796026100699.

## Table 1 Long description

This table summarises how many intersectional strata fall into ranges of individuals per stratum, out of 180 total strata. The largest share of strata (66 strata, 36.67%) have 30–99 individuals, followed by 10–29 individuals in 48 strata (26.67%), and 100–499 individuals in 31 strata (17.22%). Smaller strata (5–9 individuals) are also common (26 strata, 14.44%). Very large strata (500–999, and 1000-1310 individuals) are less common (3.33%, and 1.67%, respectively). The distribution is mostly concentrated in mid-sized strata, with few strata above 500 individuals.

Navigate back to Table 1.

## Table 2 Long description

This table reports sample sizes (n, %) and mean emotional problems scores (with SD) for OxWell 2023 participants overall, and by maltreatment status (maltreated, not maltreated, and missing maltreatment data). Emotional problems data were available for 84.22% overall (16,499/19,590), including 98.26% of the maltreated group and 98.02% of the not maltreated group. Mean emotional problems were higher among maltreated young people (14.46, SD 8.58) than among those not maltreated (8.37, SD 7.13), and the mean was 10.34 (SD 8.19) among those with missing maltreatment data. Across all maltreatment categories, females had higher mean scores than males, and individuals with “other/prefer not to say” gender identities had the highest means overall. Household poverty was associated with higher mean scores across all maltreatment categories. Mean emotional problems were broadly similar across ethnic groups, year groups, and levels of school-level deprivation.

Navigate back to Table 2.

## Table 3 Long description

This table reports predicted emotionalproblem scores (RCADS-11), with 95% approximate confidence intervals, for specific strata defined by gender, ethnicity, household poverty, school year group, and school-level deprivation, by maltreatment exposure. Across maltreatment categories,the highest predicted scores were observed for White individuals with ‘other/prefer not to say’ gender identities experiencing household poverty, and the lowest predicted scores for non-White males without poverty. Maltreatment effects (predicted differences in emotional problems by maltreatment exposure) were strongest for females or other/PNS gender identities, predominantly among those experiencing poverty. The smallest differences were observed for males, predominantly among those not experiencing poverty.

Navigate back to Table 3.

## Figure 1 Long description

The y-axis shows μ1j, and the x-axis shows the stratum rank (ordered by negative to positive deviation from the average effect). A series of points with 95% approximate confidence intervals are plotted across increasing stratum ranks. A horizontal line references μ1j = 0 The leftmost points are around μ1jminus 1.5 to minus 1.0, and the rightmost points at the positive of these values. . Significant deviations are highlighted in blue, as labelled in the legend.

Navigate back to Figure 1.

## Figure 2 Long description

The x-axis shows the stratum rank, ordered by no maltreatment exposure predicted values. The y-axis shows predicted emotional problems (RCADS-11). Two sets of points are plotted across strata, for maltreatment and no maltreatment exposure, with vertical lines connecting the paired points at each rank.

Navigate back to Figure 2.

## Table 4 Long description

Five multilevel models were used to estimate the effects of different variables on emotional problems (with 95% confidence intervals) and to derive variance components, progressing from a null model to models adding maltreatment and then additional indicators of social position. Maltreatment was consistently associated with higher emotional problems (4.46 to 4.69 across models). In Models 4–5, higher scores were also associated with female (4.57) and ‘other/prefer not to say’ gender (about 7.7), household poverty (about 4.6), and being in Years 10–11 (about 1.0). Ethnically minoritised groups had lower scores than the White group, except the MIxed ethnicity group, which was not significantly different. School-level deprivation effects were not significant. Between-stratum variance reduced from 19.59 (Model 1) to 0.38–0.46 (Models 4–5), and the VPC from 26.98% to 0.84–0.94% respectively, indicating most between-stratum differences are accounted for by the additive effects of the included social positions. Estimates should be interpreted as statistical rather than causal effects, with the corresponding uncertainty conveyed by the confidence intervals.

Navigate back to Table 4.
