# Intersectional inequalities in childhood maltreatment and adolescent emotional problems in England: a random-coefficient MAIHDA analysis – CORRIGENDUM

**DOI:** 10.1017/S2045796026100766

**Published:** 2026-07-09

**Authors:** Laura Havers, Mina Fazel, Melanie Smuk, Daisy Fancourt, Peter Fonagy, Paul McCrone, Gabriela Pavarini, Kamaldeep Bhui, Georgina M. Hosang, Sania Shakoor

Affiliation 1 contained a typographical error in the original published article. The correct affiliation is shown here.

^1^Centre for Psychiatry and Mental Health, Wolfson Institute of Population Health, Queen Mary, University of London, London, UK

The authors apologise for the error.
