# Supplementary material for: Intersectional inequalities in childhood maltreatment and adolescent emotional problems in England: a random-coefficient MAIHDA analysis
Source: Epidemiol Psychiatr Sci. 2026 Jun 4;35:e34. doi: 10.1017/S2045796026100699 (PMC13247913; doi:10.1017/S2045796026100699)
Supplement: Havers et al. supplementary material [file S2045796026100699sup001.docx]

**Supplementary Materials S1.** Deviations from preregistration

The current study deviated from the preregistration (<https://osf.io/8ryzw/overview>) in the following ways. Before examination of the data, it was decided that only pupils in school years 7-11 would be included to maintain consistency across other work from our group. It was also decided that bullying-victimisation data would not be included as an additional index of adversity. This was to focus on the effects of childhood maltreatment as specific and severe form of adversity. Upon initial examination of the data, it was decided that urbanicity would not be included as an indicator of place owing to very low cell sizes for non-urban areas. School-level variation was captured by proxy through school-level IMD.

**Supplementary Materials S2.** Emotional problems and maltreatment in excluded individuals

Among the 8,652 individuals excluded due to non-complete data on gender, ethnicity, and household poverty – mean emotional problems was *M* = 10.53 (*SD* = 8.58; *n* = 4,665), compared to *M* = 10.54 (*SD* = 8.22; *n* = 16,499) in the analytic sample. For maltreatment, the proportion of individuals reporting any experience was 35.10%, compared to 36.19% in the analytic sample.

**Supplementary Materials S3.** Emotional problems items

*Revised Child Depression and Anxiety Scale (Radez et al. 2021)*

1. I have trouble going to school in the mornings because I feel nervous or afraid
2. I have no energy for things
3. I worry when I go to bed at night
4. I worry about what is going to happen
5. Nothing is much fun anymore
6. All of a sudden, I feel really scared for no reason at all
7. I feel worthless
8. I feel sad or empty
9. When I have a problem, my heart beats really fast
10. I am tired a lot
11. I worry I might look foolish

Participant-level mean imputation was used to calculate total scores where up to one item was missing for depression (items 2, 5, 7, 8, 10) and up to one missing for anxiety (items 1, 3, 4, 6, 9, 11). This restriction was applied to preserve the scale’s measurement properties, with participants missing more than one item per subscale (and thus without a computable total score) accommodated within the broader multiple imputation framework. The non-imputed mean emotional problems was M = 10.51 (SD = 8.25; n = 15,826), compared to the analytic mean emotional problems M = 10.54 (SD = 8.22; n = 16,499).

**Supplementary Materials S4.** Childhood maltreatment

*Short Child Maltreatment Questionnaire (Meinck et al. 2016)*

1. Did a parent or other adult in the household hit, beat, kick or physically try to hurt you in any way?
2. Did a parent or other adult in the household swear at you, insult you, humiliate you, threaten you or make you feel unwanted?
3. Did someone at least five years older than you or an adult touch or fondle you or have you touch their body in a sexual way?
4. Did your parent/caregiver for long periods of time not provide you with enough food or drink, clean clothes, or a clean and warm place to live?
5. Were there times when there was no adult living with you who made you feel loved?
6. Did you see or hear one of your parents/carers being slapped, kicked, punched, beaten or deliberately hurt by a partner or ex?

Participants were allowed to give multiple (frequency) responses to the above items. In the current study, any response of “yes” (either lifetime or past 12 months) was taken to indicate exposure to maltreatment, regardless of any additional responses. Sensitivity analyses indicated that lifetime experiences of maltreatment were not substantively higher among individuals in school years 10-11, compared to school years 7-9.

**Supplementary Materials S5.** Indicators of social position

Participants were asked the following demographic questions:

- What is your year group?
- What is your gender?
- What is your ethnic group?

Response options for gender were: (i) female, (ii) male, (iii) other, (iv) prefer not to say. The ‘other’ and ‘prefer not to say’ categories were aggregated to form a single (other/PNS) category, which is further commented on in the *Discussion*.

Response options for ethnicity were: (i) White – English/Welsh/Scottish/Northern Irish/British, (ii) White – Irish (iii) White - Gypsy or Irish Traveller, (iv) White – Other, (v) Mixed/Multiple ethnic groups – White and Black Caribbean, (vi) Mixed/Multiple ethnic groups – White and Black African, (vii) Mixed/Multiple ethnic groups – White and Asian, (viii) Mixed/Multiple ethnic groups – Other, (ix) Asian/Asian British – Indian, (x) Asian/Asian British – Pakistani, (xi) Asian/Asian British – Bangladeshi, (xii) Asian/Asian British – Chinese, (xiii) Asian/Asian British – Other, (xiv) Black/African/Caribbean/Black British – African, (xv) Black/African/Caribbean/Black British – Caribbean, (xvi) Black/African/Caribbean/Black British – Other, (xvii) Arab, (xviii) Other ethnic group. Categories were aggregated into the broader groupings (White, Black, South Asian, Other Asian, Mixed, Other) – further commented on in the *Discussion*.

Participants were asked to rate the following statements about household poverty from *Wales’s Young People’s Survey on Child & Family Poverty (2019)*:

1. I worry about not having enough money for the things my family needs, e.g., food, bills, electric or gas
2. My family uses food banks
3. The house I live in is cold and/or damp
4. At school, I am unable to afford the right uniform, games kit, books, equipment, or go on trips
5. At school, I am unable to afford to eat
6. At home, I do not have enough space to do things like homework or chill out
7. At home, I have no internet access or poor internet access

And the following statement, devised by the OxWell study team:

- At home, I go to bed hungry because there is not enough food in the house

**Supplementary Materials S6.** Intersectional strata

Excluded strata with *N*<5:

- MidDeprived_Black_Y7Y9_otherPNS_NoPoverty
- LeastDeprived_Other_Y10Y11_otherPNS_NoPoverty
- MostDeprived_AsianSA_Y10Y11_otherPNS_Poverty
- MidDeprived_AsianOther_Y10Y11_otherPNS_Poverty
- MostDeprived_AsianOther_Y10Y11_otherPNS_Poverty
- MostDeprived_Black_Y10Y11_otherPNS_Poverty
- MidDeprived_Other_Y10Y11_otherPNS_Poverty
- MostDeprived_Other_Y10Y11_otherPNS_Poverty
- MostDeprived_AsianOther_Y10Y11_otherPNS_NoPoverty
- LeastDeprived_Black_Y10Y11_otherPNS_NoPoverty
- LeastDeprived_AsianSA_Y7Y9_otherPNS_Poverty
- MidDeprived_Other_Y7Y9_otherPNS_Poverty
- MostDeprived_Mixed_Y10Y11_otherPNS_Poverty
- MidDeprived_AsianSA_Y10Y11_otherPNS_Poverty
- LeastDeprived_Black_Y10Y11_otherPNS_Poverty
- MidDeprived_Black_Y10Y11_otherPNS_Poverty
- LeastDeprived_Black_Y7Y9_otherPNS_NoPoverty
- LeastDeprived_Other_Y7Y9_otherPNS_NoPoverty
- MostDeprived_AsianSA_Y10Y11_otherPNS_NoPoverty
- MidDeprived_AsianOther_Y10Y11_otherPNS_NoPoverty
- MostDeprived_Black_Y10Y11_otherPNS_NoPoverty
- MostDeprived_Other_Y10Y11_otherPNS_NoPoverty
- MostDeprived_AsianSA_Y7Y9_otherPNS_Poverty
- MidDeprived_AsianOther_Y7Y9_otherPNS_Poverty
- LeastDeprived_Black_Y7Y9_otherPNS_Poverty
- MidDeprived_Black_Y7Y9_otherPNS_Poverty
- LeastDeprived_Other_Y7Y9_otherPNS_Poverty
- LeastDeprived_AsianOther_Y10Y11_otherPNS_Poverty
- MidDeprived_AsianOther_Y7Y9_otherPNS_NoPoverty
- MostDeprived_Mixed_Y10Y11_otherPNS_NoPoverty
- MidDeprived_Black_Y10Y11_otherPNS_NoPoverty
- MidDeprived_Other_Y10Y11_otherPNS_NoPoverty
- MidDeprived_AsianSA_Y7Y9_otherPNS_Poverty
- LeastDeprived_AsianOther_Y7Y9_otherPNS_Poverty
- MidDeprived_Mixed_Y10Y11_otherPNS_Poverty
- LeastDeprived_Other_Y10Y11_otherPNS_Poverty

Stratum label order: school-level deprivation, ethnicity, school year group, gender, household poverty. AsianSA = South Asian; Other = other ethnicity groups; otherPNS = other and prefer not to say.

**Supplementary Materials S7.** Variance decomposition coefficients

Full description of coefficients and their formulas are reported in *Evans et al. (2023), p.4-5:*

**Variance Partition Coefficient (VPC):** The proportion of total variance in emotional problems attributable to between-stratum differences.

- **Model 1:** Between-stratum variance divided by total variance:

VPC = [σ²_u0_ / (σ²_u0_ + σ²_e0_)] × 100%

- **Model 2:** Residual between-stratum variance after adjusting for maltreatment as a fixed effect (single VPC value).
- **Model 3:** Residual between-stratum variance, calculated and reported separately by maltreatment exposure, when maltreatment is modelled as a random coefficient.
  - For those not exposed to maltreatment, the formula is the same as Models 1–2.
  - For those exposed to maltreatment:

VPC_maltreatment_ = [(σ²_u0_ + 2σ_u0u1_ + σ²_u1_) / (σ²_u0_ + 2σ_u0u1_ + σ²_u1_ + σ²_e0_)] × 100%

- **Model 4:** Residual between-stratum variance after adjusting for maltreatment and additive fixed effects (single VPC value).
- **Model 5:** Residual between-stratum variance when maltreatment is a random coefficient and additive fixed effects are included. VPC is calculated and reported separately by maltreatment exposure, using the same structure as in Model 3.

**Proportional Change in Variance (PCV):** The proportion of between-stratum variance explained by adding fixed effects, compared to a specified reference model.

- **Models 2 and 4 (compared to Model 1):**

PCV = [(σ²_u0_,_Model1_ − σ²_u0_,_Model2,4_) / σ²_u0_,_Model1_] × 100%

- **Model 5 (random coefficient plus additive fixed effects, relative to Model 3):**
- For those not exposed to maltreatment:

PCV_no_maltreatment_ = [(σ²_u0_,_Model3_ − σ²_u0_,_Model5_) / σ²_u0_,_Model3_] × 100%

- For those exposed to maltreatment:

PCV_maltreatment_ = [((σ²_u0_ + 2σ_u0u1_ + σ²_u1_),_Model3_ − (σ²_u0_ + 2σ_u0u1_ + σ²_u1_),_Model5_) / (σ²_u0_ + 2σ_u0u1_ + σ²_u1_),_Model3_] × 100%

**Where:**

- **σ²_u0_** = variance of the stratum-level intercept
- **σ²_u1_** = variance of the stratum-level maltreatment coefficient
- **σ_u0u1_** = covariance between the stratum-level intercept and maltreatment coefficient
- **σ²_e0_ =** individual-level residual variance (within-stratum variance)

**Supplementary Table 1.** Exposure to maltreatment for each stratum

| **Stratum** | ***n* in stratum** | ***n* with maltreatment data** | ***n* reporting exposure to maltreatment** | **Percentage with maltreatment data exposed to maltreatment** |
| --- | --- | --- | --- | --- |
| LeastDeprived_Other_Y10Y11_Male_Poverty | 6 | <10 | <10 | − |
| LeastDeprived_Other_Y10Y11_Female_Poverty | 7 | <10 | <10 | − |
| MidDeprived_Mixed_Y7Y9_otherPNS_Poverty | 5 | <10 | <10 | − |
| MostDeprived_Other_Y7Y9_otherPNS_Poverty | 7 | <10 | <10 | − |
| MidDeprived_Mixed_Y10Y11_Female_Poverty | 14 | 13 | 12 | 92.3 |
| LeastDeprived_AsianOther_Y10Y11_Male_Poverty | 15 | 12 | 11 | 91.7 |
| MidDeprived_AsianOther_Y10Y11_Female_Poverty | 10 | <10 | <10 | − |
| MidDeprived_Black_Y7Y9_Female_Poverty | 18 | <10 | <10 | − |
| LeastDeprived_Mixed_Y7Y9_otherPNS_Poverty | 9 | <10 | <10 | − |
| LeastDeprived_Black_Y10Y11_Male_Poverty | 13 | <10 | <10 | − |
| MostDeprived_Mixed_Y7Y9_otherPNS_NoPoverty | 8 | <10 | <10 | − |
| LeastDeprived_AsianOther_Y10Y11_otherPNS_NoPoverty | 7 | <10 | <10 | − |
| MostDeprived_Mixed_Y10Y11_Male_Poverty | 12 | <10 | <10 | − |
| MidDeprived_AsianOther_Y10Y11_Male_Poverty | 10 | <10 | <10 | − |
| MidDeprived_Other_Y10Y11_Male_Poverty | 13 | <10 | <10 | − |
| MostDeprived_AsianOther_Y10Y11_Female_Poverty | 14 | <10 | <10 | − |
| MostDeprived_Mixed_Y7Y9_Female_Poverty | 63 | 38 | 30 | 78.9 |
| LeastDeprived_Black_Y10Y11_Female_Poverty | 25 | 19 | 15 | 78.9 |
| MidDeprived_White_Y10Y11_otherPNS_Poverty | 15 | 14 | 11 | 78.6 |
| MidDeprived_AsianSA_Y10Y11_Female_Poverty | 27 | 18 | 14 | 77.8 |
| MidDeprived_Mixed_Y7Y9_otherPNS_NoPoverty | 9 | <10 | <10 | − |
| MostDeprived_Black_Y7Y9_otherPNS_NoPoverty | 5 | <10 | <10 | − |
| MidDeprived_Mixed_Y10Y11_otherPNS_NoPoverty | 5 | <10 | <10 | − |
| LeastDeprived_Black_Y7Y9_Female_Poverty | 29 | 24 | 18 | 75.0 |
| MidDeprived_White_Y7Y9_otherPNS_Poverty | 36 | 20 | 15 | 75.0 |
| MostDeprived_Black_Y7Y9_otherPNS_Poverty | 5 | <10 | <10 | − |
| LeastDeprived_Mixed_Y10Y11_Female_Poverty | 23 | 19 | 14 | 73.7 |
| MostDeprived_Mixed_Y10Y11_Female_Poverty | 38 | 30 | 22 | 73.3 |
| LeastDeprived_Mixed_Y7Y9_Female_Poverty | 36 | 21 | 15 | 71.4 |
| LeastDeprived_White_Y10Y11_otherPNS_Poverty | 30 | 17 | 12 | 70.6 |
| MostDeprived_White_Y10Y11_otherPNS_Poverty | 19 | 13 | <10 | − |
| MidDeprived_Black_Y10Y11_Female_Poverty | 21 | 16 | 11 | 68.8 |
| MostDeprived_AsianSA_Y10Y11_Female_Poverty | 26 | 19 | 13 | 68.4 |
| LeastDeprived_AsianOther_Y10Y11_Female_Poverty | 23 | 19 | 13 | 68.4 |
| MostDeprived_Black_Y7Y9_Female_Poverty | 53 | 34 | 23 | 67.6 |
| MostDeprived_White_Y10Y11_Female_Poverty | 204 | 148 | 100 | 67.6 |
| MidDeprived_AsianSA_Y7Y9_otherPNS_NoPoverty | 8 | <10 | <10 | − |
| MidDeprived_AsianOther_Y7Y9_Male_Poverty | 18 | <10 | <10 | − |
| MostDeprived_AsianSA_Y10Y11_Male_Poverty | 15 | <10 | <10 | − |
| LeastDeprived_White_Y10Y11_Female_Poverty | 193 | 140 | 93 | 66.4 |
| LeastDeprived_White_Y7Y9_otherPNS_Poverty | 59 | 44 | 29 | 65.9 |
| MostDeprived_Mixed_Y7Y9_Male_Poverty | 22 | 14 | <10 | − |
| MostDeprived_White_Y7Y9_otherPNS_Poverty | 62 | 44 | 28 | 63.6 |
| MidDeprived_White_Y10Y11_Female_Poverty | 110 | 87 | 55 | 63.2 |
| MidDeprived_AsianOther_Y10Y11_Male_NoPoverty | 32 | 24 | 15 | 62.5 |
| MidDeprived_AsianSA_Y10Y11_otherPNS_NoPoverty | 10 | <10 | <10 | − |
| LeastDeprived_Mixed_Y10Y11_otherPNS_Poverty | 8 | <10 | <10 | − |
| MostDeprived_Black_Y10Y11_Female_Poverty | 36 | 26 | 16 | 61.5 |
| MidDeprived_White_Y7Y9_Female_Poverty | 198 | 150 | 92 | 61.3 |
| LeastDeprived_White_Y7Y9_Female_Poverty | 394 | 281 | 170 | 60.5 |
| LeastDeprived_Mixed_Y10Y11_otherPNS_NoPoverty | 7 | <10 | <10 | − |
| LeastDeprived_Black_Y7Y9_Male_Poverty | 14 | 10 | <10 | − |
| MidDeprived_Mixed_Y10Y11_Male_Poverty | 19 | 15 | <10 | − |
| MidDeprived_Black_Y10Y11_Male_Poverty | 9 | <10 | <10 | − |
| MostDeprived_AsianSA_Y7Y9_Female_Poverty | 45 | 35 | 21 | 60.0 |
| MostDeprived_AsianOther_Y7Y9_otherPNS_Poverty | 6 | <10 | <10 | − |
| LeastDeprived_AsianSA_Y10Y11_otherPNS_Poverty | 8 | <10 | <10 | − |
| MidDeprived_AsianOther_Y7Y9_Female_Poverty | 18 | 17 | 10 | 58.8 |
| MidDeprived_AsianSA_Y7Y9_Male_Poverty | 48 | 29 | 17 | 58.6 |
| LeastDeprived_AsianOther_Y7Y9_Male_Poverty | 32 | 19 | 11 | 57.9 |
| LeastDeprived_AsianSA_Y10Y11_Female_Poverty | 22 | 19 | 11 | 57.9 |
| LeastDeprived_AsianOther_Y7Y9_otherPNS_NoPoverty | 8 | <10 | <10 | − |
| MostDeprived_Mixed_Y7Y9_otherPNS_Poverty | 9 | <10 | <10 | − |
| MidDeprived_White_Y10Y11_Male_Poverty | 112 | 86 | 48 | 55.8 |
| MidDeprived_Other_Y7Y9_Female_Poverty | 14 | <10 | <10 | − |
| MidDeprived_Mixed_Y7Y9_Male_Poverty | 27 | 11 | <10 | − |
| MostDeprived_Other_Y10Y11_Male_Poverty | 17 | 11 | <10 | − |
| MidDeprived_Black_Y7Y9_Male_Poverty | 22 | 13 | <10 | − |
| MidDeprived_White_Y10Y11_otherPNS_NoPoverty | 32 | 28 | 15 | 53.6 |
| LeastDeprived_White_Y7Y9_Male_Poverty | 328 | 212 | 113 | 53.3 |
| LeastDeprived_AsianSA_Y7Y9_Female_Poverty | 70 | 58 | 30 | 51.7 |
| MostDeprived_Other_Y7Y9_Female_Poverty | 72 | 43 | 22 | 51.2 |
| MostDeprived_White_Y10Y11_Male_Poverty | 87 | 61 | 31 | 50.8 |
| MidDeprived_Black_Y7Y9_Male_NoPoverty | 88 | 50 | 25 | 50.0 |
| LeastDeprived_Mixed_Y10Y11_Female_NoPoverty | 66 | 56 | 28 | 50.0 |
| MostDeprived_AsianOther_Y7Y9_otherPNS_NoPoverty | 10 | <10 | <10 | − |
| MidDeprived_AsianSA_Y10Y11_Male_Poverty | 25 | 14 | <10 | − |
| MostDeprived_Black_Y10Y11_Male_Poverty | 17 | 10 | <10 | − |
| MidDeprived_Other_Y10Y11_Female_Poverty | 6 | <10 | <10 | − |
| MostDeprived_White_Y7Y9_Male_Poverty | 257 | 181 | 90 | 49.7 |
| MostDeprived_White_Y7Y9_Female_Poverty | 408 | 270 | 134 | 49.6 |
| LeastDeprived_Black_Y10Y11_Female_NoPoverty | 61 | 44 | 21 | 47.7 |
| LeastDeprived_Other_Y7Y9_Male_Poverty | 30 | 21 | 10 | 47.6 |
| MidDeprived_Mixed_Y10Y11_Female_NoPoverty | 41 | 38 | 18 | 47.4 |
| MidDeprived_Mixed_Y7Y9_Female_Poverty | 28 | 19 | <10 | − |
| MostDeprived_AsianOther_Y7Y9_Male_Poverty | 23 | 17 | <10 | − |
| MidDeprived_White_Y7Y9_Male_Poverty | 258 | 184 | 84 | 45.7 |
| LeastDeprived_Mixed_Y7Y9_Male_Poverty | 37 | 22 | 10 | 45.5 |
| MostDeprived_AsianOther_Y7Y9_Female_Poverty | 35 | 22 | 10 | 45.5 |
| MidDeprived_Other_Y10Y11_Female_NoPoverty | 11 | <10 | <10 | − |
| LeastDeprived_White_Y10Y11_Female_NoPoverty | 658 | 453 | 200 | 44.2 |
| LeastDeprived_White_Y10Y11_Male_Poverty | 174 | 105 | 46 | 43.8 |
| MidDeprived_Other_Y7Y9_Male_Poverty | 20 | 16 | <10 | − |
| MostDeprived_AsianOther_Y10Y11_Female_NoPoverty | 50 | 39 | 17 | 43.6 |
| MidDeprived_AsianSA_Y7Y9_Female_Poverty | 45 | 30 | 13 | 43.3 |
| MostDeprived_Mixed_Y7Y9_Male_NoPoverty | 63 | 35 | 15 | 42.9 |
| LeastDeprived_Mixed_Y7Y9_otherPNS_NoPoverty | 7 | <10 | <10 | − |
| MostDeprived_AsianOther_Y10Y11_Male_Poverty | 11 | <10 | <10 | − |
| LeastDeprived_Other_Y7Y9_Female_Poverty | 26 | 14 | <10 | − |
| MostDeprived_Other_Y10Y11_Female_Poverty | 19 | 14 | <10 | − |
| MidDeprived_Mixed_Y7Y9_Male_NoPoverty | 74 | 54 | 23 | 42.6 |
| MidDeprived_Mixed_Y7Y9_Female_NoPoverty | 82 | 54 | 23 | 42.6 |
| MostDeprived_Black_Y7Y9_Male_Poverty | 35 | 19 | <10 | − |
| LeastDeprived_AsianSA_Y10Y11_Male_Poverty | 17 | 12 | <10 | − |
| MostDeprived_AsianSA_Y10Y11_Male_NoPoverty | 34 | 17 | <10 | − |
| LeastDeprived_Other_Y10Y11_Female_NoPoverty | 24 | 17 | <10 | − |
| MostDeprived_AsianSA_Y7Y9_Male_Poverty | 35 | 22 | <10 | − |
| LeastDeprived_AsianOther_Y10Y11_Female_NoPoverty | 43 | 32 | 13 | 40.6 |
| MidDeprived_Mixed_Y10Y11_Male_NoPoverty | 53 | 40 | 16 | 40.0 |
| MidDeprived_Black_Y10Y11_Female_NoPoverty | 25 | 20 | <10 | − |
| MostDeprived_Mixed_Y10Y11_Female_NoPoverty | 69 | 48 | 19 | 39.6 |
| LeastDeprived_Mixed_Y10Y11_Male_NoPoverty | 71 | 46 | 18 | 39.1 |
| MostDeprived_White_Y7Y9_otherPNS_NoPoverty | 87 | 69 | 27 | 39.1 |
| MidDeprived_AsianOther_Y7Y9_Female_NoPoverty | 98 | 77 | 30 | 39.0 |
| MostDeprived_AsianOther_Y7Y9_Male_NoPoverty | 49 | 36 | 14 | 38.9 |
| LeastDeprived_Mixed_Y10Y11_Male_Poverty | 24 | 18 | <10 | − |
| MidDeprived_Black_Y7Y9_Female_NoPoverty | 68 | 44 | 17 | 38.6 |
| MostDeprived_Other_Y7Y9_Male_Poverty | 51 | 29 | 11 | 37.9 |
| MidDeprived_Black_Y10Y11_Male_NoPoverty | 43 | 24 | <10 | − |
| MidDeprived_White_Y10Y11_Female_NoPoverty | 338 | 281 | 105 | 37.4 |
| LeastDeprived_Mixed_Y7Y9_Female_NoPoverty | 149 | 106 | 39 | 36.8 |
| LeastDeprived_AsianSA_Y7Y9_Female_NoPoverty | 313 | 251 | 92 | 36.7 |
| MidDeprived_AsianSA_Y10Y11_Female_NoPoverty | 130 | 110 | 39 | 35.5 |
| MostDeprived_AsianOther_Y7Y9_Female_NoPoverty | 94 | 79 | 28 | 35.4 |
| LeastDeprived_AsianOther_Y7Y9_Female_Poverty | 25 | 17 | <10 | − |
| LeastDeprived_Mixed_Y7Y9_Male_NoPoverty | 108 | 83 | 29 | 34.9 |
| LeastDeprived_AsianOther_Y7Y9_Female_NoPoverty | 110 | 83 | 29 | 34.9 |
| LeastDeprived_White_Y10Y11_otherPNS_NoPoverty | 73 | 52 | 18 | 34.6 |
| LeastDeprived_White_Y7Y9_otherPNS_NoPoverty | 107 | 82 | 28 | 34.1 |
| MostDeprived_Black_Y7Y9_Male_NoPoverty | 102 | 62 | 21 | 33.9 |
| LeastDeprived_Other_Y10Y11_Male_NoPoverty | 30 | 18 | <10 | − |
| MostDeprived_Other_Y10Y11_Male_NoPoverty | 36 | 21 | <10 | − |
| LeastDeprived_AsianSA_Y7Y9_Male_Poverty | 44 | 33 | 11 | 33.3 |
| MostDeprived_White_Y10Y11_Female_NoPoverty | 578 | 423 | 140 | 33.1 |
| MostDeprived_Mixed_Y7Y9_Female_NoPoverty | 142 | 103 | 34 | 33.0 |
| MostDeprived_AsianSA_Y7Y9_Male_NoPoverty | 95 | 67 | 22 | 32.8 |
| LeastDeprived_Black_Y7Y9_Male_NoPoverty | 73 | 40 | 13 | 32.5 |
| MidDeprived_White_Y7Y9_otherPNS_NoPoverty | 47 | 34 | 11 | 32.4 |
| MostDeprived_Black_Y7Y9_Female_NoPoverty | 159 | 113 | 36 | 31.9 |
| LeastDeprived_AsianOther_Y10Y11_Male_NoPoverty | 69 | 44 | 14 | 31.8 |
| MostDeprived_AsianOther_Y10Y11_Male_NoPoverty | 31 | 22 | <10 | − |
| LeastDeprived_Black_Y7Y9_Female_NoPoverty | 79 | 54 | 17 | 31.5 |
| LeastDeprived_AsianSA_Y10Y11_Female_NoPoverty | 135 | 112 | 35 | 31.2 |
| MidDeprived_AsianSA_Y10Y11_Male_NoPoverty | 73 | 49 | 15 | 30.6 |
| LeastDeprived_White_Y7Y9_Female_NoPoverty | 1310 | 980 | 292 | 29.8 |
| LeastDeprived_Black_Y10Y11_Male_NoPoverty | 30 | 17 | <10 | − |
| MidDeprived_White_Y10Y11_Male_NoPoverty | 394 | 301 | 86 | 28.6 |
| LeastDeprived_White_Y10Y11_Male_NoPoverty | 738 | 444 | 126 | 28.4 |
| LeastDeprived_Other_Y7Y9_Male_NoPoverty | 83 | 53 | 15 | 28.3 |
| LeastDeprived_AsianSA_Y7Y9_Male_NoPoverty | 244 | 174 | 49 | 28.2 |
| MidDeprived_White_Y7Y9_Female_NoPoverty | 753 | 560 | 155 | 27.7 |
| LeastDeprived_White_Y7Y9_Male_NoPoverty | 1266 | 870 | 240 | 27.6 |
| MidDeprived_AsianSA_Y7Y9_Female_NoPoverty | 252 | 212 | 58 | 27.4 |
| MostDeprived_AsianSA_Y10Y11_Female_NoPoverty | 89 | 55 | 15 | 27.3 |
| LeastDeprived_AsianOther_Y7Y9_Male_NoPoverty | 130 | 85 | 23 | 27.1 |
| MostDeprived_Other_Y7Y9_Female_NoPoverty | 152 | 105 | 28 | 26.7 |
| MostDeprived_White_Y7Y9_Female_NoPoverty | 1277 | 954 | 247 | 25.9 |
| MidDeprived_Other_Y7Y9_otherPNS_NoPoverty | 7 | <10 | <10 | − |
| MostDeprived_White_Y10Y11_otherPNS_NoPoverty | 28 | 20 | <10 | − |
| LeastDeprived_AsianSA_Y10Y11_otherPNS_NoPoverty | 5 | <10 | <10 | − |
| MidDeprived_White_Y7Y9_Male_NoPoverty | 965 | 714 | 172 | 24.1 |
| MostDeprived_Black_Y10Y11_Male_NoPoverty | 46 | 25 | <10 | − |
| LeastDeprived_Other_Y7Y9_Female_NoPoverty | 54 | 34 | <10 | − |
| MostDeprived_White_Y10Y11_Male_NoPoverty | 349 | 226 | 53 | 23.5 |
| MostDeprived_Other_Y10Y11_Female_NoPoverty | 57 | 35 | <10 | − |
| MostDeprived_White_Y7Y9_Male_NoPoverty | 817 | 589 | 129 | 21.9 |
| MidDeprived_Other_Y7Y9_Male_NoPoverty | 39 | 24 | <10 | − |
| MidDeprived_AsianSA_Y7Y9_Male_NoPoverty | 195 | 129 | 26 | 20.2 |
| MostDeprived_AsianSA_Y7Y9_Female_NoPoverty | 187 | 134 | 27 | 20.1 |
| MostDeprived_Mixed_Y10Y11_Male_NoPoverty | 26 | 15 | <10 | − |
| LeastDeprived_AsianSA_Y7Y9_otherPNS_NoPoverty | 9 | <10 | <10 | − |
| MidDeprived_AsianOther_Y10Y11_Female_NoPoverty | 36 | 31 | <10 | − |
| MidDeprived_Other_Y7Y9_Female_NoPoverty | 31 | 21 | <10 | − |
| MostDeprived_Black_Y10Y11_Female_NoPoverty | 69 | 49 | <10 | − |
| MostDeprived_Other_Y7Y9_Male_NoPoverty | 103 | 70 | 11 | 15.7 |
| LeastDeprived_AsianSA_Y10Y11_Male_NoPoverty | 97 | 70 | 10 | 14.3 |
| MidDeprived_Other_Y10Y11_Male_NoPoverty | 23 | 16 | <10 | − |
| MidDeprived_AsianOther_Y7Y9_Male_NoPoverty | 70 | 51 | <10 | − |
| MostDeprived_AsianSA_Y7Y9_otherPNS_NoPoverty | 6 | <10 | <10 | − |
| MostDeprived_Other_Y7Y9_otherPNS_NoPoverty | 6 | <10 | <10 | − |

*Note*. Emotional problems indexed by the Revised Child Depression and Anxiety Scale (RCADS-11). Stratum label order: school-level deprivation, ethnicity, school year group, gender, household poverty. AsianSA = South Asian; Other = other ethnicity groups; otherPNS = other and prefer not to say. Cell counts <10 and associated percentages are suppressed to minimise disclosure risk.

**Supplementary Table 2.** Predicted stratum-level emotional problems for individuals exposed to maltreatment

| **Stratum** | **Predicted emotional problems** | ***SE*** | **CI Low** | **CI High** |
| --- | --- | --- | --- | --- |
| LeastDeprived_White_Y10Y11_otherPNS_Poverty | 24.48 | 0.85 | 22.82 | 26.14 |
| MostDeprived_White_Y10Y11_otherPNS_Poverty | 24.20 | 0.65 | 22.92 | 25.47 |
| MostDeprived_Mixed_Y7Y9_otherPNS_Poverty | 23.62 | 1.41 | 20.86 | 26.38 |
| MostDeprived_White_Y7Y9_otherPNS_Poverty | 22.63 | 0.41 | 21.82 | 23.44 |
| MostDeprived_Black_Y7Y9_otherPNS_Poverty | 22.49 | 0.26 | 21.98 | 23.01 |
| MidDeprived_White_Y10Y11_otherPNS_Poverty | 21.67 | 0.09 | 21.50 | 21.84 |
| LeastDeprived_White_Y10Y11_Female_Poverty | 21.55 | 0.34 | 20.88 | 22.22 |
| MidDeprived_White_Y10Y11_Female_Poverty | 21.33 | 0.44 | 20.46 | 22.20 |
| LeastDeprived_White_Y7Y9_otherPNS_Poverty | 21.19 | 0.52 | 20.17 | 22.20 |
| MidDeprived_White_Y7Y9_otherPNS_Poverty | 20.99 | 0.87 | 19.29 | 22.69 |
| MostDeprived_AsianSA_Y7Y9_Female_Poverty | 20.88 | 0.45 | 20.00 | 21.77 |
| MidDeprived_White_Y7Y9_Female_Poverty | 20.21 | 0.32 | 19.58 | 20.84 |
| LeastDeprived_Mixed_Y10Y11_otherPNS_Poverty | 20.13 | 0.12 | 19.90 | 20.36 |
| LeastDeprived_AsianOther_Y10Y11_otherPNS_NoPoverty | 19.96 | 0.31 | 19.35 | 20.56 |
| LeastDeprived_White_Y10Y11_otherPNS_NoPoverty | 19.92 | 0.58 | 18.79 | 21.05 |
| LeastDeprived_White_Y7Y9_Female_Poverty | 19.84 | 0.19 | 19.47 | 20.20 |
| MidDeprived_AsianSA_Y10Y11_Female_Poverty | 19.63 | 0.54 | 18.57 | 20.69 |
| LeastDeprived_Mixed_Y7Y9_otherPNS_NoPoverty | 19.58 | 0.15 | 19.28 | 19.87 |
| MostDeprived_Other_Y7Y9_otherPNS_Poverty | 19.47 | 0.46 | 18.57 | 20.37 |
| MostDeprived_White_Y10Y11_Female_Poverty | 19.37 | 0.26 | 18.87 | 19.87 |
| MostDeprived_White_Y7Y9_Female_Poverty | 19.36 | 0.39 | 18.60 | 20.12 |
| MostDeprived_Black_Y10Y11_Female_Poverty | 19.33 | 0.95 | 17.47 | 21.20 |
| LeastDeprived_AsianSA_Y10Y11_otherPNS_NoPoverty | 19.05 | 1.19 | 16.72 | 21.39 |
| LeastDeprived_Mixed_Y10Y11_Female_Poverty | 19.03 | 1.05 | 16.97 | 21.08 |
| LeastDeprived_AsianSA_Y10Y11_otherPNS_Poverty | 19.00 | 1.44 | 16.19 | 21.82 |
| MostDeprived_White_Y10Y11_otherPNS_NoPoverty | 18.98 | 1.20 | 16.63 | 21.33 |
| MostDeprived_Mixed_Y10Y11_Female_Poverty | 18.94 | 0.59 | 17.78 | 20.10 |
| LeastDeprived_AsianOther_Y10Y11_Female_Poverty | 18.91 | 0.39 | 18.16 | 19.67 |
| MidDeprived_Mixed_Y7Y9_Female_Poverty | 18.82 | 1.15 | 16.57 | 21.08 |
| MidDeprived_Mixed_Y10Y11_Female_Poverty | 18.76 | 0.14 | 18.48 | 19.04 |
| MostDeprived_Other_Y7Y9_Female_Poverty | 18.59 | 0.64 | 17.34 | 19.84 |
| MidDeprived_White_Y7Y9_otherPNS_NoPoverty | 18.53 | 0.93 | 16.71 | 20.34 |
| LeastDeprived_Black_Y7Y9_Female_Poverty | 18.40 | 0.44 | 17.54 | 19.26 |
| MidDeprived_Mixed_Y7Y9_otherPNS_Poverty | 18.29 | 1.07 | 16.21 | 20.38 |
| MostDeprived_AsianOther_Y10Y11_Female_Poverty | 18.20 | 1.00 | 16.25 | 20.15 |
| LeastDeprived_AsianOther_Y7Y9_otherPNS_NoPoverty | 18.18 | 0.46 | 17.29 | 19.07 |
| MostDeprived_AsianOther_Y7Y9_Female_Poverty | 18.01 | 1.12 | 15.81 | 20.20 |
| MostDeprived_Mixed_Y7Y9_Female_Poverty | 17.91 | 0.62 | 16.70 | 19.11 |
| MostDeprived_AsianSA_Y10Y11_Female_Poverty | 17.82 | 0.84 | 16.17 | 19.47 |
| MidDeprived_AsianOther_Y10Y11_Female_Poverty | 17.79 | 1.12 | 15.59 | 19.98 |
| MidDeprived_Black_Y10Y11_Female_Poverty | 17.73 | 0.82 | 16.13 | 19.33 |
| MostDeprived_Other_Y10Y11_Female_Poverty | 17.72 | 0.93 | 15.90 | 19.53 |
| LeastDeprived_Other_Y10Y11_Female_Poverty | 17.61 | 1.58 | 14.52 | 20.71 |
| MidDeprived_Mixed_Y10Y11_Female_NoPoverty | 17.61 | 0.53 | 16.57 | 18.65 |
| MidDeprived_AsianSA_Y7Y9_Female_Poverty | 17.53 | 0.65 | 16.26 | 18.81 |
| LeastDeprived_White_Y7Y9_otherPNS_NoPoverty | 17.51 | 0.41 | 16.70 | 18.31 |
| MostDeprived_Mixed_Y7Y9_otherPNS_NoPoverty | 17.46 | 1.30 | 14.91 | 20.02 |
| MidDeprived_AsianOther_Y7Y9_Female_Poverty | 17.42 | 0.08 | 17.25 | 17.58 |
| MidDeprived_White_Y10Y11_otherPNS_NoPoverty | 17.41 | 0.71 | 16.01 | 18.80 |
| LeastDeprived_AsianSA_Y10Y11_Female_Poverty | 17.29 | 0.74 | 15.85 | 18.73 |
| MostDeprived_White_Y7Y9_otherPNS_NoPoverty | 17.29 | 0.43 | 16.45 | 18.12 |
| LeastDeprived_AsianSA_Y7Y9_Female_Poverty | 17.28 | 0.46 | 16.38 | 18.17 |
| MostDeprived_Mixed_Y10Y11_Male_Poverty | 17.27 | 1.09 | 15.13 | 19.41 |
| LeastDeprived_Mixed_Y7Y9_otherPNS_Poverty | 17.24 | 0.99 | 15.30 | 19.19 |
| LeastDeprived_Mixed_Y7Y9_Female_Poverty | 17.15 | 0.67 | 15.83 | 18.47 |
| MostDeprived_AsianOther_Y7Y9_otherPNS_Poverty | 16.97 | 0.47 | 16.06 | 17.88 |
| MostDeprived_Black_Y7Y9_Female_Poverty | 16.94 | 0.48 | 16.01 | 17.87 |
| LeastDeprived_AsianOther_Y7Y9_Female_Poverty | 16.79 | 1.15 | 14.54 | 19.04 |
| MidDeprived_Other_Y7Y9_Female_Poverty | 16.79 | 0.92 | 14.99 | 18.58 |
| LeastDeprived_Mixed_Y10Y11_Male_Poverty | 16.79 | 0.53 | 15.74 | 17.83 |
| LeastDeprived_Black_Y7Y9_Male_Poverty | 16.75 | 1.10 | 14.59 | 18.91 |
| MidDeprived_AsianSA_Y10Y11_otherPNS_NoPoverty | 16.68 | 1.31 | 14.11 | 19.24 |
| LeastDeprived_Other_Y7Y9_Male_Poverty | 16.55 | 0.79 | 15.01 | 18.10 |
| LeastDeprived_Other_Y7Y9_Female_Poverty | 16.45 | 1.12 | 14.25 | 18.64 |
| MostDeprived_AsianOther_Y7Y9_otherPNS_NoPoverty | 16.35 | 1.40 | 13.61 | 19.09 |
| MidDeprived_White_Y10Y11_Female_NoPoverty | 16.29 | 0.29 | 15.72 | 16.85 |
| MidDeprived_Other_Y10Y11_Female_NoPoverty | 16.27 | 0.69 | 14.92 | 17.62 |
| MostDeprived_AsianSA_Y10Y11_Male_Poverty | 16.20 | 1.32 | 13.61 | 18.78 |
| LeastDeprived_White_Y10Y11_Female_NoPoverty | 15.73 | 0.29 | 15.17 | 16.29 |
| MostDeprived_AsianSA_Y10Y11_Female_NoPoverty | 15.68 | 0.52 | 14.66 | 16.70 |
| LeastDeprived_Black_Y10Y11_Female_Poverty | 15.66 | 1.24 | 13.23 | 18.10 |
| LeastDeprived_White_Y7Y9_Female_NoPoverty | 15.64 | 0.31 | 15.04 | 16.24 |
| MostDeprived_White_Y10Y11_Male_Poverty | 15.61 | 0.41 | 14.82 | 16.41 |
| MidDeprived_Mixed_Y7Y9_otherPNS_NoPoverty | 15.57 | 0.33 | 14.92 | 16.23 |
| MidDeprived_Other_Y10Y11_Female_Poverty | 15.53 | 0.08 | 15.37 | 15.69 |
| MidDeprived_Black_Y7Y9_Female_Poverty | 15.45 | 1.81 | 11.91 | 19.00 |
| LeastDeprived_Mixed_Y10Y11_Female_NoPoverty | 15.43 | 0.39 | 14.67 | 16.20 |
| MidDeprived_AsianOther_Y10Y11_Female_NoPoverty | 15.08 | 0.46 | 14.17 | 15.98 |
| LeastDeprived_AsianOther_Y10Y11_Male_Poverty | 15.05 | 0.84 | 13.41 | 16.69 |
| MostDeprived_Black_Y7Y9_otherPNS_NoPoverty | 15.01 | 0.40 | 14.22 | 15.80 |
| MostDeprived_AsianOther_Y10Y11_Female_NoPoverty | 14.94 | 0.46 | 14.05 | 15.83 |
| MostDeprived_Mixed_Y10Y11_Female_NoPoverty | 14.82 | 0.74 | 13.38 | 16.27 |
| LeastDeprived_Mixed_Y7Y9_Male_Poverty | 14.82 | 1.01 | 12.85 | 16.79 |
| MostDeprived_White_Y10Y11_Female_NoPoverty | 14.73 | 0.29 | 14.16 | 15.30 |
| MidDeprived_Mixed_Y10Y11_Male_Poverty | 14.71 | 1.07 | 12.61 | 16.81 |
| MostDeprived_White_Y7Y9_Female_NoPoverty | 14.64 | 0.25 | 14.15 | 15.13 |
| MostDeprived_Other_Y7Y9_otherPNS_NoPoverty | 14.63 | 0.47 | 13.70 | 15.56 |
| MidDeprived_White_Y7Y9_Female_NoPoverty | 14.63 | 0.26 | 14.12 | 15.13 |
| LeastDeprived_AsianSA_Y7Y9_Male_Poverty | 14.49 | 0.88 | 12.76 | 16.22 |
| LeastDeprived_Mixed_Y7Y9_Female_NoPoverty | 14.34 | 0.47 | 13.43 | 15.26 |
| LeastDeprived_AsianSA_Y10Y11_Female_NoPoverty | 14.27 | 0.43 | 13.44 | 15.11 |
| MidDeprived_Other_Y10Y11_Male_Poverty | 14.24 | 1.19 | 11.91 | 16.57 |
| MidDeprived_AsianSA_Y10Y11_Male_Poverty | 14.23 | 0.77 | 12.73 | 15.74 |
| MidDeprived_AsianOther_Y7Y9_Male_Poverty | 14.12 | 1.21 | 11.75 | 16.50 |
| MidDeprived_White_Y7Y9_Male_Poverty | 14.01 | 0.31 | 13.40 | 14.61 |
| LeastDeprived_White_Y10Y11_Male_Poverty | 13.99 | 0.55 | 12.91 | 15.06 |
| MostDeprived_Other_Y10Y11_Female_NoPoverty | 13.97 | 0.83 | 12.35 | 15.60 |
| MidDeprived_AsianSA_Y10Y11_Female_NoPoverty | 13.91 | 0.36 | 13.20 | 14.62 |
| LeastDeprived_AsianSA_Y7Y9_Female_NoPoverty | 13.78 | 0.20 | 13.39 | 14.17 |
| MidDeprived_Mixed_Y7Y9_Male_Poverty | 13.69 | 1.24 | 11.27 | 16.11 |
| MostDeprived_Black_Y10Y11_Male_Poverty | 13.66 | 0.99 | 11.72 | 15.60 |
| LeastDeprived_AsianSA_Y7Y9_otherPNS_NoPoverty | 13.66 | 2.52 | 8.71 | 18.60 |
| MostDeprived_White_Y7Y9_Male_Poverty | 13.65 | 0.49 | 12.69 | 14.61 |
| LeastDeprived_Black_Y10Y11_Female_NoPoverty | 13.60 | 0.64 | 12.35 | 14.85 |
| LeastDeprived_White_Y7Y9_Male_Poverty | 13.57 | 0.25 | 13.08 | 14.05 |
| MidDeprived_AsianSA_Y7Y9_otherPNS_NoPoverty | 13.46 | 0.91 | 11.67 | 15.25 |
| LeastDeprived_AsianSA_Y10Y11_Male_Poverty | 13.39 | 1.00 | 11.43 | 15.35 |
| MostDeprived_Mixed_Y7Y9_Female_NoPoverty | 13.37 | 0.34 | 12.70 | 14.04 |
| MostDeprived_AsianOther_Y7Y9_Male_Poverty | 13.37 | 0.94 | 11.53 | 15.22 |
| MostDeprived_Other_Y7Y9_Male_Poverty | 13.37 | 0.89 | 11.63 | 15.11 |
| MostDeprived_AsianSA_Y7Y9_otherPNS_NoPoverty | 13.36 | 1.27 | 10.86 | 15.85 |
| LeastDeprived_Other_Y10Y11_Female_NoPoverty | 13.15 | 0.84 | 11.51 | 14.79 |
| LeastDeprived_Black_Y10Y11_Male_Poverty | 13.07 | 1.68 | 9.77 | 16.37 |
| LeastDeprived_AsianOther_Y7Y9_Female_NoPoverty | 13.06 | 0.55 | 11.98 | 14.15 |
| MostDeprived_Black_Y10Y11_Female_NoPoverty | 13.00 | 0.45 | 12.13 | 13.88 |
| LeastDeprived_Other_Y10Y11_Male_Poverty | 12.95 | 1.39 | 10.22 | 15.68 |
| MidDeprived_AsianSA_Y7Y9_Female_NoPoverty | 12.93 | 0.22 | 12.50 | 13.36 |
| MostDeprived_AsianOther_Y7Y9_Female_NoPoverty | 12.80 | 0.40 | 12.02 | 13.58 |
| MostDeprived_Black_Y7Y9_Female_Poverty | 12.78 | 0.42 | 11.95 | 13.62 |
| LeastDeprived_Mixed_Y10Y11_otherPNS_NoPoverty | 12.78 | 1.05 | 10.71 | 14.84 |
| LeastDeprived_Other_Y7Y9_Female_NoPoverty | 12.77 | 0.92 | 10.97 | 14.57 |
| LeastDeprived_Black_Y7Y9_Female_NoPoverty | 12.68 | 0.57 | 11.56 | 13.80 |
| MidDeprived_White_Y10Y11_Male_Poverty | 12.68 | 0.35 | 11.99 | 13.36 |
| MidDeprived_AsianSA_Y7Y9_Male_Poverty | 12.61 | 0.78 | 11.08 | 14.13 |
| MostDeprived_AsianSA_Y7Y9_Female_NoPoverty | 12.53 | 0.45 | 11.64 | 13.42 |
| MostDeprived_Other_Y7Y9_Female_NoPoverty | 12.53 | 0.49 | 11.56 | 13.50 |
| MidDeprived_Mixed_Y7Y9_Female_NoPoverty | 12.44 | 0.48 | 11.49 | 13.39 |
| MostDeprived_Black_Y7Y9_Male_Poverty | 12.33 | 0.97 | 10.43 | 14.22 |
| MidDeprived_Other_Y7Y9_otherPNS_NoPoverty | 12.23 | 2.31 | 7.70 | 16.77 |
| MidDeprived_AsianOther_Y7Y9_Female_NoPoverty | 12.21 | 0.36 | 11.52 | 12.91 |
| MostDeprived_Mixed_Y7Y9_Male_Poverty | 12.15 | 0.97 | 10.24 | 14.06 |
| MidDeprived_AsianOther_Y10Y11_Male_Poverty | 12.09 | 1.66 | 8.83 | 15.35 |
| MostDeprived_AsianOther_Y10Y11_Male_Poverty | 12.07 | 0.84 | 10.42 | 13.72 |
| LeastDeprived_AsianOther_Y10Y11_Female_NoPoverty | 11.90 | 0.63 | 10.67 | 13.13 |
| MidDeprived_Other_Y10Y11_Male_NoPoverty | 11.89 | 0.79 | 10.33 | 13.45 |
| MostDeprived_AsianSA_Y7Y9_Male_Poverty | 11.77 | 0.71 | 10.38 | 13.17 |
| MidDeprived_Mixed_Y10Y11_otherPNS_NoPoverty | 11.75 | 1.35 | 9.11 | 14.39 |
| MostDeprived_Other_Y10Y11_Male_Poverty | 11.55 | 1.08 | 9.44 | 13.67 |
| LeastDeprived_AsianOther_Y7Y9_Male_NoPoverty | 11.48 | 0.57 | 10.36 | 12.61 |
| MidDeprived_Other_Y7Y9_Female_NoPoverty | 11.37 | 1.08 | 9.25 | 13.48 |
| MidDeprived_Black_Y10Y11_Male_Poverty | 11.37 | 1.02 | 9.36 | 13.37 |
| MidDeprived_Black_Y10Y11_Female_NoPoverty | 11.26 | 0.46 | 10.35 | 12.17 |
| MidDeprived_Black_Y7Y9_Male_Poverty | 11.24 | 1.06 | 9.15 | 13.32 |
| MidDeprived_Black_Y7Y9_Female_NoPoverty | 11.18 | 0.41 | 10.39 | 11.98 |
| MostDeprived_AsianOther_Y10Y11_Male_NoPoverty | 11.05 | 1.14 | 8.81 | 13.29 |
| LeastDeprived_Black_Y10Y11_Male_NoPoverty | 11.00 | 1.02 | 8.99 | 13.00 |
| MidDeprived_Other_Y7Y9_Male_NoPoverty | 10.93 | 0.76 | 9.45 | 12.41 |
| MidDeprived_AsianOther_Y7Y9_Male_NoPoverty | 10.85 | 0.59 | 9.70 | 12.00 |
| LeastDeprived_AsianSA_Y10Y11_Male_NoPoverty | 10.56 | 0.62 | 9.35 | 11.77 |
| MidDeprived_AsianOther_Y10Y11_Male_NoPoverty | 10.47 | 0.84 | 8.83 | 12.11 |
| LeastDeprived_Mixed_Y10Y11_Male_NoPoverty | 10.29 | 0.72 | 8.87 | 11.70 |
| MidDeprived_White_Y10Y11_Male_NoPoverty | 10.16 | 0.36 | 9.45 | 10.87 |
| MostDeprived_Mixed_Y7Y9_Male_NoPoverty | 10.15 | 0.85 | 8.48 | 11.81 |
| MostDeprived_Mixed_Y10Y11_Male_NoPoverty | 10.14 | 1.17 | 7.86 | 12.43 |
| MidDeprived_Black_Y10Y11_Male_NoPoverty | 10.10 | 0.83 | 8.48 | 11.73 |
| LeastDeprived_AsianOther_Y10Y11_Male_NoPoverty | 10.10 | 0.78 | 8.57 | 11.63 |
| MostDeprived_Other_Y7Y9_Male_NoPoverty | 9.78 | 0.55 | 8.69 | 10.86 |
| MostDeprived_White_Y10Y11_Male_NoPoverty | 9.71 | 0.50 | 8.73 | 10.69 |
| MostDeprived_White_Y7Y9_Male_NoPoverty | 9.71 | 0.33 | 9.06 | 10.36 |
| LeastDeprived_Mixed_Y7Y9_Male_NoPoverty | 9.59 | 0.35 | 8.91 | 10.28 |
| MostDeprived_AsianOther_Y7Y9_Male_NoPoverty | 9.51 | 0.48 | 8.56 | 10.45 |
| LeastDeprived_White_Y10Y11_Male_NoPoverty | 9.50 | 0.24 | 9.03 | 9.97 |
| MidDeprived_Other_Y7Y9_Male_NoPoverty | 9.21 | 0.82 | 7.61 | 10.81 |
| MidDeprived_Mixed_Y10Y11_Male_NoPoverty | 9.17 | 0.57 | 8.06 | 10.28 |
| MidDeprived_White_Y7Y9_Male_NoPoverty | 9.14 | 0.26 | 8.64 | 9.65 |
| MidDeprived_AsianSA_Y10Y11_Male_NoPoverty | 9.08 | 0.59 | 7.92 | 10.24 |
| LeastDeprived_AsianOther_Y7Y9_Male_NoPoverty | 8.96 | 0.57 | 7.84 | 10.07 |
| LeastDeprived_White_Y7Y9_Male_NoPoverty | 8.95 | 0.24 | 8.47 | 9.42 |
| MidDeprived_Mixed_Y7Y9_Male_NoPoverty | 8.76 | 0.70 | 7.39 | 10.13 |
| LeastDeprived_AsianSA_Y7Y9_Male_NoPoverty | 8.63 | 0.46 | 7.72 | 9.53 |
| LeastDeprived_Other_Y10Y11_Male_NoPoverty | 8.53 | 0.89 | 6.78 | 10.28 |
| MidDeprived_AsianSA_Y7Y9_Male_NoPoverty | 8.38 | 0.49 | 7.41 | 9.35 |
| MostDeprived_AsianSA_Y10Y11_Male_NoPoverty | 8.31 | 1.07 | 6.21 | 10.41 |
| MostDeprived_Black_Y7Y9_Male_NoPoverty | 8.19 | 0.43 | 7.35 | 9.04 |
| LeastDeprived_Other_Y7Y9_Male_NoPoverty | 8.10 | 0.78 | 6.57 | 9.62 |
| MostDeprived_Other_Y10Y11_Male_NoPoverty | 8.06 | 1.05 | 6.01 | 10.12 |
| MostDeprived_AsianSA_Y7Y9_Male_NoPoverty | 8.05 | 0.61 | 6.85 | 9.25 |
| MostDeprived_Black_Y10Y11_Male_NoPoverty | 7.85 | 0.79 | 6.29 | 9.40 |
| LeastDeprived_Black_Y7Y9_Male_NoPoverty | 7.72 | 0.91 | 5.93 | 9.51 |
| MidDeprived_Black_Y7Y9_Male_NoPoverty | 7.57 | 0.61 | 6.39 | 8.76 |

*Note*. Emotional problems indexed by the Revised Child Depression and Anxiety Scale (RCADS-11). 95% approximate CI. Stratum label order: school-level deprivation, ethnicity, school year group, gender, household poverty. AsianSA = South Asian; Other = other ethnicity groups; otherPNS = other and prefer not to say. Estimates derived from Model 3.

**Supplementary Table 3.** Predicted stratum-level emotional problems for individuals not exposed to maltreatment

| **Stratum** | **Predicted emotional problems** | ***SE*** | **CI Low** | **CI High** |
| --- | --- | --- | --- | --- |
| LeastDeprived_White_Y10Y11_otherPNS_Poverty | 18.58 | 0.93 | 16.77 | 20.40 |
| MostDeprived_White_Y10Y11_otherPNS_Poverty | 18.25 | 0.64 | 17.01 | 19.50 |
| MostDeprived_Mixed_Y7Y9_otherPNS_Poverty | 17.79 | 1.31 | 15.23 | 20.35 |
| MostDeprived_White_Y7Y9_otherPNS_Poverty | 17.18 | 0.48 | 16.25 | 18.12 |
| MostDeprived_Black_Y7Y9_otherPNS_Poverty | 16.78 | 0.41 | 15.97 | 17.59 |
| LeastDeprived_White_Y10Y11_Female_Poverty | 16.37 | 0.40 | 15.58 | 17.16 |
| MidDeprived_White_Y10Y11_Female_Poverty | 16.27 | 0.34 | 15.61 | 16.93 |
| MidDeprived_White_Y10Y11_otherPNS_Poverty | 16.09 | 0.28 | 15.53 | 16.64 |
| LeastDeprived_White_Y7Y9_otherPNS_Poverty | 15.74 | 0.44 | 14.89 | 16.60 |
| MidDeprived_White_Y7Y9_otherPNS_Poverty | 15.55 | 0.88 | 13.83 | 17.27 |
| MidDeprived_White_Y7Y9_Female_Poverty | 14.95 | 0.34 | 14.28 | 15.62 |
| MostDeprived_White_Y10Y11_Female_Poverty | 14.83 | 0.42 | 14.01 | 15.65 |
| MostDeprived_AsianSA_Y7Y9_Female_Poverty | 14.81 | 0.43 | 13.97 | 15.65 |
| LeastDeprived_Mixed_Y10Y11_otherPNS_Poverty | 14.69 | 0.18 | 14.32 | 15.05 |
| LeastDeprived_White_Y7Y9_Female_Poverty | 14.52 | 0.33 | 13.88 | 15.16 |
| LeastDeprived_AsianOther_Y10Y11_otherPNS_NoPoverty | 14.45 | 0.38 | 13.70 | 15.20 |
| LeastDeprived_White_Y10Y11_otherPNS_NoPoverty | 14.41 | 0.45 | 13.53 | 15.29 |
| MidDeprived_AsianSA_Y10Y11_Female_Poverty | 14.38 | 0.53 | 13.34 | 15.43 |
| LeastDeprived_Mixed_Y7Y9_otherPNS_NoPoverty | 14.20 | 0.14 | 13.92 | 14.47 |
| MostDeprived_Black_Y10Y11_Female_Poverty | 14.17 | 0.90 | 12.41 | 15.93 |
| MostDeprived_Other_Y7Y9_otherPNS_Poverty | 14.05 | 0.37 | 13.32 | 14.78 |
| MostDeprived_White_Y7Y9_Female_Poverty | 13.80 | 0.38 | 13.05 | 14.55 |
| MostDeprived_White_Y10Y11_otherPNS_NoPoverty | 13.79 | 1.12 | 11.60 | 15.98 |
| LeastDeprived_AsianSA_Y10Y11_otherPNS_NoPoverty | 13.77 | 1.06 | 11.69 | 15.86 |
| LeastDeprived_Mixed_Y10Y11_Female_Poverty | 13.77 | 0.93 | 11.95 | 15.58 |
| MostDeprived_Mixed_Y10Y11_Female_Poverty | 13.73 | 0.57 | 12.61 | 14.86 |
| LeastDeprived_AsianOther_Y10Y11_Female_Poverty | 13.67 | 0.35 | 12.99 | 14.34 |
| LeastDeprived_AsianSA_Y10Y11_otherPNS_Poverty | 13.65 | 1.14 | 11.41 | 15.89 |
| MidDeprived_Mixed_Y10Y11_Female_Poverty | 13.63 | 0.35 | 12.93 | 14.32 |
| MidDeprived_Mixed_Y7Y9_Female_Poverty | 13.36 | 0.95 | 11.49 | 15.22 |
| MostDeprived_Other_Y7Y9_Female_Poverty | 13.23 | 0.51 | 12.24 | 14.23 |
| MostDeprived_AsianOther_Y10Y11_Female_Poverty | 13.14 | 0.75 | 11.66 | 14.62 |
| MostDeprived_AsianSA_Y10Y11_Female_Poverty | 13.06 | 0.88 | 11.34 | 14.78 |
| MidDeprived_Mixed_Y7Y9_otherPNS_Poverty | 13.02 | 0.87 | 11.31 | 14.73 |
| MidDeprived_White_Y7Y9_otherPNS_NoPoverty | 12.97 | 0.75 | 11.50 | 14.45 |
| LeastDeprived_Black_Y7Y9_Female_Poverty | 12.91 | 0.26 | 12.40 | 13.43 |
| LeastDeprived_AsianOther_Y7Y9_otherPNS_NoPoverty | 12.90 | 0.26 | 12.38 | 13.41 |
| MostDeprived_AsianOther_Y7Y9_Female_Poverty | 12.77 | 0.93 | 10.95 | 14.59 |
| MostDeprived_Mixed_Y7Y9_Female_Poverty | 12.74 | 0.55 | 11.67 | 13.81 |
| MidDeprived_AsianOther_Y10Y11_Female_Poverty | 12.68 | 1.02 | 10.67 | 14.68 |
| MidDeprived_White_Y10Y11_otherPNS_NoPoverty | 12.67 | 0.67 | 11.36 | 13.97 |
| MidDeprived_AsianSA_Y7Y9_Female_Poverty | 12.60 | 0.39 | 11.83 | 13.36 |
| MidDeprived_Black_Y10Y11_Female_Poverty | 12.56 | 0.69 | 11.21 | 13.92 |
| LeastDeprived_Other_Y10Y11_Female_Poverty | 12.49 | 1.39 | 9.76 | 15.22 |
| MostDeprived_Other_Y10Y11_Female_Poverty | 12.49 | 0.87 | 10.78 | 14.20 |
| MostDeprived_Mixed_Y7Y9_otherPNS_NoPoverty | 12.48 | 1.29 | 9.96 | 15.00 |
| LeastDeprived_AsianSA_Y10Y11_Female_Poverty | 12.33 | 0.74 | 10.87 | 13.78 |
| MidDeprived_AsianOther_Y7Y9_Female_Poverty | 12.31 | 0.19 | 11.95 | 12.68 |
| MidDeprived_Mixed_Y10Y11_Female_NoPoverty | 12.31 | 0.52 | 11.29 | 13.34 |
| MostDeprived_Black_Y7Y9_Female_Poverty | 12.23 | 0.47 | 11.31 | 13.15 |
| MostDeprived_Mixed_Y10Y11_Male_Poverty | 12.21 | 0.97 | 10.29 | 14.12 |
| LeastDeprived_Mixed_Y7Y9_Female_Poverty | 12.18 | 0.58 | 11.05 | 13.32 |
| MostDeprived_White_Y7Y9_otherPNS_NoPoverty | 12.09 | 0.43 | 11.26 | 12.93 |
| LeastDeprived_Mixed_Y7Y9_otherPNS_Poverty | 12.09 | 0.83 | 10.45 | 13.72 |
| LeastDeprived_AsianSA_Y7Y9_Female_Poverty | 12.08 | 0.45 | 11.20 | 12.97 |
| MostDeprived_AsianOther_Y7Y9_otherPNS_Poverty | 12.02 | 0.35 | 11.34 | 12.70 |
| LeastDeprived_AsianOther_Y7Y9_Female_Poverty | 12.01 | 0.89 | 10.26 | 13.76 |
| LeastDeprived_White_Y7Y9_otherPNS_NoPoverty | 11.99 | 0.44 | 11.13 | 12.85 |
| MidDeprived_White_Y10Y11_Female_NoPoverty | 11.78 | 0.20 | 11.39 | 12.18 |
| LeastDeprived_Black_Y7Y9_Male_Poverty | 11.77 | 0.89 | 10.03 | 13.51 |
| MidDeprived_AsianSA_Y10Y11_otherPNS_NoPoverty | 11.76 | 1.22 | 9.36 | 14.15 |
| MidDeprived_Other_Y7Y9_Female_Poverty | 11.74 | 0.88 | 10.02 | 13.46 |
| LeastDeprived_Other_Y7Y9_Female_Poverty | 11.52 | 0.87 | 9.82 | 13.23 |
| LeastDeprived_Mixed_Y10Y11_Male_Poverty | 11.48 | 0.43 | 10.64 | 12.31 |
| MostDeprived_AsianSA_Y10Y11_Male_Poverty | 11.38 | 1.04 | 9.35 | 13.42 |
| MostDeprived_AsianOther_Y7Y9_otherPNS_NoPoverty | 11.36 | 1.18 | 9.05 | 13.67 |
| LeastDeprived_White_Y10Y11_Female_NoPoverty | 11.32 | 0.27 | 10.80 | 11.84 |
| LeastDeprived_Other_Y7Y9_Male_Poverty | 11.29 | 0.72 | 9.88 | 12.71 |
| MidDeprived_Other_Y10Y11_Female_NoPoverty | 11.24 | 0.59 | 10.08 | 12.40 |
| LeastDeprived_Mixed_Y10Y11_Female_NoPoverty | 11.22 | 0.37 | 10.50 | 11.95 |
| LeastDeprived_Black_Y10Y11_Female_Poverty | 11.07 | 1.10 | 8.90 | 13.23 |
| MostDeprived_AsianSA_Y10Y11_Female_NoPoverty | 10.88 | 0.48 | 9.93 | 11.82 |
| MidDeprived_Mixed_Y7Y9_otherPNS_NoPoverty | 10.84 | 0.33 | 10.19 | 11.49 |
| MidDeprived_Other_Y10Y11_Female_Poverty | 10.82 | 0.13 | 10.57 | 11.07 |
| MidDeprived_Black_Y7Y9_Female_Poverty | 10.72 | 1.67 | 7.44 | 14.00 |
| MidDeprived_AsianOther_Y10Y11_Female_NoPoverty | 10.45 | 0.36 | 9.74 | 11.17 |
| LeastDeprived_AsianOther_Y10Y11_Male_Poverty | 10.33 | 0.66 | 9.04 | 11.63 |
| MidDeprived_Mixed_Y10Y11_Male_Poverty | 10.32 | 1.08 | 8.21 | 12.43 |
| MostDeprived_Mixed_Y10Y11_Female_NoPoverty | 10.28 | 0.59 | 9.11 | 11.44 |
| MostDeprived_White_Y10Y11_Male_Poverty | 10.26 | 0.32 | 9.63 | 10.88 |
| MostDeprived_Black_Y7Y9_otherPNS_NoPoverty | 10.20 | 0.31 | 9.59 | 10.80 |
| MostDeprived_White_Y7Y9_Male_Poverty | 10.07 | 0.41 | 9.26 | 10.88 |
| LeastDeprived_Mixed_Y7Y9_Male_Poverty | 10.00 | 0.84 | 8.35 | 11.65 |
| MostDeprived_Other_Y7Y9_otherPNS_NoPoverty | 9.96 | 0.37 | 9.24 | 10.68 |
| MostDeprived_AsianOther_Y10Y11_Female_NoPoverty | 9.82 | 0.38 | 9.07 | 10.56 |
| MostDeprived_White_Y10Y11_Female_NoPoverty | 9.77 | 0.20 | 9.38 | 10.16 |
| LeastDeprived_Mixed_Y7Y9_Female_NoPoverty | 9.77 | 0.41 | 8.97 | 10.56 |
| LeastDeprived_AsianSA_Y7Y9_Male_Poverty | 9.76 | 0.65 | 8.49 | 11.02 |
| MidDeprived_White_Y7Y9_Female_NoPoverty | 9.67 | 0.13 | 9.42 | 9.92 |
| MostDeprived_Other_Y10Y11_Female_NoPoverty | 9.63 | 0.61 | 8.43 | 10.82 |
| LeastDeprived_White_Y7Y9_Female_NoPoverty | 9.50 | 0.12 | 9.26 | 9.74 |
| MidDeprived_Other_Y10Y11_Male_Poverty | 9.46 | 0.97 | 7.55 | 11.36 |
| LeastDeprived_White_Y10Y11_Male_Poverty | 9.45 | 0.52 | 8.42 | 10.48 |
| LeastDeprived_White_Y7Y9_Male_Poverty | 9.36 | 0.36 | 8.65 | 10.08 |
| MidDeprived_AsianSA_Y10Y11_Male_Poverty | 9.33 | 0.70 | 7.95 | 10.70 |
| MidDeprived_AsianOther_Y7Y9_Male_Poverty | 9.28 | 1.12 | 7.09 | 11.47 |
| LeastDeprived_AsianSA_Y10Y11_Female_NoPoverty | 9.26 | 0.26 | 8.75 | 9.78 |
| LeastDeprived_Black_Y10Y11_Female_NoPoverty | 9.22 | 0.60 | 8.04 | 10.39 |
| MidDeprived_White_Y7Y9_Male_Poverty | 9.19 | 0.33 | 8.55 | 9.84 |
| MidDeprived_AsianSA_Y10Y11_Female_NoPoverty | 9.10 | 0.21 | 8.69 | 9.51 |
| MostDeprived_AsianOther_Y7Y9_Male_Poverty | 9.07 | 0.96 | 7.19 | 10.94 |
| MostDeprived_White_Y7Y9_Female_NoPoverty | 9.05 | 0.09 | 8.88 | 9.23 |
| MostDeprived_Other_Y7Y9_Male_Poverty | 9.04 | 0.60 | 7.86 | 10.21 |
| LeastDeprived_AsianSA_Y7Y9_otherPNS_NoPoverty | 9.00 | 2.14 | 4.80 | 13.20 |
| MidDeprived_Mixed_Y7Y9_Male_Poverty | 8.98 | 1.16 | 6.70 | 11.26 |
| MostDeprived_Black_Y10Y11_Male_Poverty | 8.96 | 0.79 | 7.41 | 10.51 |
| MostDeprived_AsianSA_Y7Y9_otherPNS_NoPoverty | 8.84 | 1.09 | 6.70 | 10.97 |
| LeastDeprived_AsianSA_Y10Y11_Male_Poverty | 8.83 | 0.74 | 7.38 | 10.29 |
| MidDeprived_AsianSA_Y7Y9_otherPNS_NoPoverty | 8.83 | 0.81 | 7.23 | 10.43 |
| LeastDeprived_AsianOther_Y7Y9_Female_NoPoverty | 8.81 | 0.36 | 8.09 | 9.52 |
| LeastDeprived_Other_Y10Y11_Female_NoPoverty | 8.80 | 0.74 | 7.34 | 10.25 |
| MostDeprived_Mixed_Y7Y9_Female_NoPoverty | 8.76 | 0.36 | 8.06 | 9.46 |
| MostDeprived_AsianOther_Y7Y9_Female_NoPoverty | 8.74 | 0.31 | 8.14 | 9.35 |
| MostDeprived_Black_Y10Y11_Female_NoPoverty | 8.63 | 0.44 | 7.77 | 9.50 |
| MidDeprived_White_Y10Y11_Male_Poverty | 8.56 | 0.28 | 8.02 | 9.11 |
| LeastDeprived_Black_Y10Y11_Male_Poverty | 8.54 | 1.52 | 5.56 | 11.51 |
| LeastDeprived_Mixed_Y10Y11_otherPNS_NoPoverty | 8.53 | 0.96 | 6.65 | 10.41 |
| LeastDeprived_Other_Y10Y11_Male_Poverty | 8.52 | 1.17 | 6.23 | 10.82 |
| LeastDeprived_Other_Y7Y9_Female_NoPoverty | 8.46 | 0.79 | 6.90 | 10.01 |
| MostDeprived_Other_Y7Y9_Female_NoPoverty | 8.32 | 0.34 | 7.66 | 8.99 |
| LeastDeprived_AsianSA_Y7Y9_Female_NoPoverty | 8.30 | 0.27 | 7.76 | 8.83 |
| MidDeprived_AsianSA_Y7Y9_Male_Poverty | 8.30 | 0.60 | 7.12 | 9.47 |
| MidDeprived_AsianSA_Y7Y9_Female_NoPoverty | 8.01 | 0.18 | 7.66 | 8.36 |
| LeastDeprived_AsianOther_Y10Y11_Female_NoPoverty | 7.98 | 0.51 | 6.98 | 8.97 |
| MostDeprived_AsianOther_Y10Y11_Male_Poverty | 7.95 | 0.76 | 6.45 | 9.44 |
| MidDeprived_Mixed_Y7Y9_Female_NoPoverty | 7.93 | 0.44 | 7.08 | 8.79 |
| LeastDeprived_Black_Y7Y9_Female_NoPoverty | 7.92 | 0.48 | 6.98 | 8.86 |
| MidDeprived_Other_Y7Y9_otherPNS_NoPoverty | 7.84 | 2.06 | 3.80 | 11.88 |
| MostDeprived_AsianSA_Y7Y9_Female_NoPoverty | 7.77 | 0.26 | 7.26 | 8.28 |
| MidDeprived_AsianOther_Y7Y9_Female_NoPoverty | 7.77 | 0.30 | 7.17 | 8.36 |
| MostDeprived_Black_Y7Y9_Male_Poverty | 7.77 | 0.85 | 6.09 | 9.44 |
| MidDeprived_AsianOther_Y10Y11_Male_Poverty | 7.71 | 1.46 | 4.86 | 10.57 |
| MostDeprived_Mixed_Y7Y9_Male_Poverty | 7.71 | 0.85 | 6.04 | 9.38 |
| MostDeprived_Black_Y7Y9_Female_NoPoverty | 7.70 | 0.27 | 7.16 | 8.23 |
| MostDeprived_AsianSA_Y7Y9_Male_Poverty | 7.65 | 0.50 | 6.67 | 8.63 |
| MidDeprived_Other_Y10Y11_Male_NoPoverty | 7.51 | 0.75 | 6.04 | 8.99 |
| MidDeprived_Mixed_Y10Y11_otherPNS_NoPoverty | 7.51 | 1.22 | 5.13 | 9.89 |
| LeastDeprived_AsianOther_Y7Y9_Male_Poverty | 7.51 | 0.54 | 6.44 | 8.57 |
| MostDeprived_Other_Y10Y11_Male_Poverty | 7.37 | 1.00 | 5.41 | 9.33 |
| MidDeprived_Black_Y10Y11_Female_NoPoverty | 7.28 | 0.38 | 6.53 | 8.03 |
| MidDeprived_Other_Y7Y9_Female_NoPoverty | 7.26 | 0.91 | 5.48 | 9.05 |
| MidDeprived_Black_Y7Y9_Male_Poverty | 7.25 | 0.91 | 5.46 | 9.04 |
| MidDeprived_Black_Y10Y11_Male_Poverty | 7.15 | 0.75 | 5.67 | 8.62 |
| MidDeprived_AsianOther_Y7Y9_Male_NoPoverty | 6.92 | 0.43 | 6.08 | 7.76 |
| MidDeprived_Other_Y7Y9_Male_Poverty | 6.69 | 0.70 | 5.33 | 8.06 |
| MostDeprived_AsianOther_Y10Y11_Male_NoPoverty | 6.63 | 1.02 | 4.63 | 8.62 |
| LeastDeprived_Black_Y10Y11_Male_NoPoverty | 6.55 | 0.79 | 5.00 | 8.11 |
| LeastDeprived_AsianSA_Y10Y11_Male_NoPoverty | 6.47 | 0.40 | 5.69 | 7.25 |
| MidDeprived_Black_Y7Y9_Female_NoPoverty | 6.39 | 0.19 | 6.01 | 6.76 |
| MidDeprived_AsianOther_Y10Y11_Male_NoPoverty | 6.26 | 0.73 | 4.82 | 7.69 |
| LeastDeprived_Mixed_Y10Y11_Male_NoPoverty | 6.23 | 0.61 | 5.04 | 7.43 |
| MidDeprived_Black_Y10Y11_Male_NoPoverty | 6.10 | 0.69 | 4.75 | 7.44 |
| LeastDeprived_AsianOther_Y10Y11_Male_NoPoverty | 6.09 | 0.67 | 4.78 | 7.40 |
| LeastDeprived_White_Y10Y11_Male_NoPoverty | 6.09 | 0.22 | 5.65 | 6.52 |
| MostDeprived_Mixed_Y10Y11_Male_NoPoverty | 6.08 | 0.95 | 4.21 | 7.94 |
| MidDeprived_White_Y10Y11_Male_NoPoverty | 5.97 | 0.22 | 5.54 | 6.40 |
| LeastDeprived_Mixed_Y7Y9_Male_NoPoverty | 5.89 | 0.28 | 5.33 | 6.44 |
| MostDeprived_Mixed_Y7Y9_Male_NoPoverty | 5.86 | 0.63 | 4.63 | 7.10 |
| MostDeprived_White_Y7Y9_Male_NoPoverty | 5.85 | 0.16 | 5.55 | 6.16 |
| MostDeprived_Other_Y7Y9_Male_NoPoverty | 5.56 | 0.44 | 4.71 | 6.42 |
| LeastDeprived_White_Y7Y9_Male_NoPoverty | 5.49 | 0.12 | 5.25 | 5.74 |
| MidDeprived_Other_Y7Y9_Male_NoPoverty | 5.49 | 0.68 | 4.16 | 6.82 |
| MostDeprived_White_Y10Y11_Male_NoPoverty | 5.47 | 0.28 | 4.92 | 6.02 |
| MidDeprived_Mixed_Y10Y11_Male_NoPoverty | 5.44 | 0.45 | 4.57 | 6.32 |
| MostDeprived_AsianOther_Y7Y9_Male_NoPoverty | 5.33 | 0.39 | 4.57 | 6.10 |
| MidDeprived_AsianSA_Y10Y11_Male_NoPoverty | 5.15 | 0.46 | 4.25 | 6.06 |
| MidDeprived_White_Y7Y9_Male_NoPoverty | 5.02 | 0.12 | 4.80 | 5.25 |
| MostDeprived_Black_Y7Y9_Male_NoPoverty | 4.99 | 0.49 | 4.03 | 5.96 |
| LeastDeprived_Other_Y10Y11_Male_NoPoverty | 4.77 | 0.81 | 3.19 | 6.35 |
| LeastDeprived_AsianSA_Y7Y9_Male_NoPoverty | 4.76 | 0.30 | 4.17 | 5.34 |
| LeastDeprived_AsianOther_Y7Y9_Male_NoPoverty | 4.70 | 0.39 | 3.94 | 5.47 |
| MidDeprived_AsianSA_Y7Y9_Male_NoPoverty | 4.62 | 0.37 | 3.90 | 5.34 |
| MostDeprived_AsianSA_Y10Y11_Male_NoPoverty | 4.54 | 0.93 | 2.71 | 6.37 |
| MidDeprived_Mixed_Y7Y9_Male_NoPoverty | 4.46 | 0.53 | 3.42 | 5.49 |
| MostDeprived_Other_Y10Y11_Male_NoPoverty | 4.37 | 0.86 | 2.69 | 6.05 |
| LeastDeprived_Other_Y7Y9_Male_NoPoverty | 4.30 | 0.65 | 3.03 | 5.57 |
| LeastDeprived_Black_Y7Y9_Male_NoPoverty | 4.24 | 0.67 | 2.94 | 5.55 |
| MostDeprived_AsianSA_Y7Y9_Male_NoPoverty | 4.09 | 0.36 | 3.39 | 4.79 |
| MostDeprived_Black_Y10Y11_Male_NoPoverty | 4.01 | 0.73 | 2.57 | 5.44 |
| MidDeprived_Black_Y7Y9_Male_NoPoverty | 3.79 | 0.66 | 2.50 | 5.09 |

*Note*. Emotional problems indexed by the Revised Child Depression and Anxiety Scale (RCADS-11). 95% approximate CI. Stratum label order: school-level deprivation, ethnicity, school year group, gender, household poverty. AsianSA = South Asian; Other = other ethnicity groups; otherPNS = other and prefer not to say. Estimates derived from Model 3.

**Supplementary Table 4.** Predicted stratum-level maltreatment effects (*β_1j_*) on emotional problems

| **Cluster** | **Predicted difference in emotional problems** (*β_1j_*) | ***SE*** | **CI Low** | **CI High** |
| --- | --- | --- | --- | --- |
| LeastDeprived_White_Y7Y9_Female_NoPoverty | 6.14 | 0.34 | 5.48 | 6.80 |
| MostDeprived_AsianSA_Y7Y9_Female_Poverty | 6.07 | 0.24 | 5.60 | 6.55 |
| MostDeprived_White_Y10Y11_otherPNS_Poverty | 5.94 | 0.49 | 4.97 | 6.91 |
| LeastDeprived_White_Y10Y11_otherPNS_Poverty | 5.89 | 0.48 | 4.95 | 6.84 |
| MostDeprived_Mixed_Y7Y9_otherPNS_Poverty | 5.83 | 0.42 | 5.00 | 6.66 |
| MostDeprived_Black_Y7Y9_otherPNS_Poverty | 5.71 | 0.37 | 4.99 | 6.44 |
| MostDeprived_White_Y7Y9_Female_NoPoverty | 5.59 | 0.27 | 5.05 | 6.12 |
| MidDeprived_White_Y10Y11_otherPNS_Poverty | 5.58 | 0.34 | 4.92 | 6.25 |
| MostDeprived_White_Y7Y9_Female_Poverty | 5.57 | 0.47 | 4.64 | 6.49 |
| MidDeprived_White_Y7Y9_otherPNS_NoPoverty | 5.55 | 0.33 | 4.91 | 6.19 |
| LeastDeprived_White_Y7Y9_otherPNS_NoPoverty | 5.52 | 0.27 | 5.00 | 6.04 |
| LeastDeprived_White_Y10Y11_otherPNS_NoPoverty | 5.51 | 0.46 | 4.60 | 6.42 |
| LeastDeprived_AsianOther_Y10Y11_otherPNS_NoPoverty | 5.50 | 0.27 | 4.97 | 6.03 |
| LeastDeprived_Black_Y7Y9_Female_Poverty | 5.49 | 0.28 | 4.94 | 6.03 |
| LeastDeprived_AsianSA_Y7Y9_Female_NoPoverty | 5.48 | 0.24 | 5.02 | 5.95 |
| MidDeprived_Mixed_Y7Y9_Female_Poverty | 5.47 | 0.41 | 4.67 | 6.26 |
| MostDeprived_White_Y7Y9_otherPNS_Poverty | 5.45 | 0.45 | 4.56 | 6.34 |
| LeastDeprived_Mixed_Y10Y11_otherPNS_Poverty | 5.44 | 0.29 | 4.88 | 6.01 |
| LeastDeprived_White_Y7Y9_otherPNS_Poverty | 5.44 | 0.42 | 4.62 | 6.26 |
| MidDeprived_White_Y7Y9_otherPNS_Poverty | 5.44 | 0.44 | 4.58 | 6.30 |
| MostDeprived_Other_Y7Y9_otherPNS_Poverty | 5.42 | 0.29 | 4.86 | 5.99 |
| LeastDeprived_Mixed_Y7Y9_otherPNS_NoPoverty | 5.38 | 0.28 | 4.84 | 5.92 |
| MostDeprived_White_Y10Y11_Male_Poverty | 5.36 | 0.35 | 4.68 | 6.04 |
| MostDeprived_Other_Y7Y9_Female_Poverty | 5.36 | 0.44 | 4.50 | 6.21 |
| LeastDeprived_AsianSA_Y10Y11_otherPNS_Poverty | 5.35 | 0.41 | 4.54 | 6.16 |
| LeastDeprived_White_Y7Y9_Female_Poverty | 5.32 | 0.37 | 4.60 | 6.05 |
| LeastDeprived_Mixed_Y10Y11_Male_Poverty | 5.31 | 0.21 | 4.89 | 5.73 |
| MidDeprived_Mixed_Y10Y11_Female_NoPoverty | 5.30 | 0.20 | 4.91 | 5.68 |
| LeastDeprived_AsianSA_Y10Y11_otherPNS_NoPoverty | 5.28 | 0.30 | 4.70 | 5.87 |
| LeastDeprived_AsianOther_Y7Y9_otherPNS_NoPoverty | 5.28 | 0.28 | 4.74 | 5.82 |
| MidDeprived_Mixed_Y7Y9_otherPNS_Poverty | 5.27 | 0.31 | 4.67 | 5.88 |
| LeastDeprived_Mixed_Y10Y11_Female_Poverty | 5.26 | 0.34 | 4.60 | 5.92 |
| LeastDeprived_Other_Y7Y9_Male_Poverty | 5.26 | 0.26 | 4.74 | 5.78 |
| MidDeprived_White_Y7Y9_Female_Poverty | 5.26 | 0.48 | 4.33 | 6.19 |
| LeastDeprived_AsianOther_Y10Y11_Female_Poverty | 5.25 | 0.26 | 4.74 | 5.76 |
| MidDeprived_AsianSA_Y10Y11_Female_Poverty | 5.24 | 0.31 | 4.65 | 5.84 |
| MostDeprived_AsianOther_Y7Y9_Female_Poverty | 5.24 | 0.41 | 4.42 | 6.05 |
| MostDeprived_Other_Y10Y11_Female_Poverty | 5.22 | 0.26 | 4.72 | 5.73 |
| MostDeprived_Mixed_Y10Y11_Female_Poverty | 5.21 | 0.25 | 4.72 | 5.69 |
| LeastDeprived_AsianSA_Y7Y9_Female_Poverty | 5.19 | 0.30 | 4.60 | 5.79 |
| MostDeprived_White_Y7Y9_otherPNS_NoPoverty | 5.19 | 0.27 | 4.66 | 5.72 |
| MostDeprived_White_Y10Y11_otherPNS_NoPoverty | 5.19 | 0.31 | 4.59 | 5.80 |
| LeastDeprived_White_Y10Y11_Female_Poverty | 5.18 | 0.46 | 4.28 | 6.07 |
| MostDeprived_Mixed_Y7Y9_Female_Poverty | 5.17 | 0.36 | 4.47 | 5.87 |
| MidDeprived_Black_Y10Y11_Female_Poverty | 5.17 | 0.29 | 4.61 | 5.73 |
| MostDeprived_Black_Y10Y11_Female_Poverty | 5.16 | 0.33 | 4.52 | 5.81 |
| LeastDeprived_Mixed_Y7Y9_otherPNS_Poverty | 5.16 | 0.27 | 4.62 | 5.69 |
| MidDeprived_Mixed_Y10Y11_Female_Poverty | 5.13 | 0.28 | 4.58 | 5.68 |
| MostDeprived_AsianOther_Y10Y11_Female_NoPoverty | 5.12 | 0.20 | 4.72 | 5.52 |
| LeastDeprived_Other_Y10Y11_Female_Poverty | 5.12 | 0.30 | 4.53 | 5.70 |
| MidDeprived_AsianOther_Y10Y11_Female_Poverty | 5.11 | 0.27 | 4.58 | 5.64 |
| MidDeprived_AsianOther_Y7Y9_Female_Poverty | 5.10 | 0.23 | 4.65 | 5.55 |
| MostDeprived_Black_Y7Y9_Female_NoPoverty | 5.09 | 0.35 | 4.40 | 5.77 |
| MostDeprived_Mixed_Y10Y11_Male_Poverty | 5.07 | 0.40 | 4.28 | 5.86 |
| MidDeprived_White_Y10Y11_Female_Poverty | 5.06 | 0.40 | 4.27 | 5.85 |
| MostDeprived_AsianOther_Y10Y11_Female_Poverty | 5.06 | 0.46 | 4.16 | 5.96 |
| MidDeprived_Other_Y7Y9_Female_Poverty | 5.05 | 0.31 | 4.44 | 5.66 |
| MidDeprived_Other_Y10Y11_Female_NoPoverty | 5.03 | 0.21 | 4.62 | 5.43 |
| LeastDeprived_AsianSA_Y10Y11_Female_NoPoverty | 5.01 | 0.26 | 4.50 | 5.52 |
| MostDeprived_AsianOther_Y7Y9_otherPNS_NoPoverty | 4.99 | 0.33 | 4.35 | 5.63 |
| MostDeprived_Mixed_Y7Y9_otherPNS_NoPoverty | 4.99 | 0.25 | 4.49 | 5.48 |
| LeastDeprived_Black_Y7Y9_Male_Poverty | 4.98 | 0.31 | 4.38 | 5.58 |
| LeastDeprived_Mixed_Y7Y9_Female_Poverty | 4.96 | 0.33 | 4.32 | 5.61 |
| LeastDeprived_AsianSA_Y10Y11_Female_Poverty | 4.96 | 0.30 | 4.37 | 5.55 |
| MostDeprived_White_Y10Y11_Female_NoPoverty | 4.96 | 0.31 | 4.36 | 5.56 |
| MidDeprived_White_Y7Y9_Female_NoPoverty | 4.95 | 0.30 | 4.37 | 5.53 |
| MostDeprived_AsianOther_Y7Y9_otherPNS_Poverty | 4.95 | 0.25 | 4.47 | 5.43 |
| MidDeprived_AsianSA_Y7Y9_Female_Poverty | 4.94 | 0.43 | 4.10 | 5.78 |
| LeastDeprived_Other_Y7Y9_Female_Poverty | 4.92 | 0.36 | 4.22 | 5.62 |
| MidDeprived_AsianSA_Y7Y9_Female_NoPoverty | 4.92 | 0.18 | 4.57 | 5.27 |
| MidDeprived_AsianSA_Y10Y11_otherPNS_NoPoverty | 4.92 | 0.25 | 4.42 | 5.42 |
| MidDeprived_AsianSA_Y10Y11_Male_Poverty | 4.91 | 0.25 | 4.42 | 5.39 |
| MidDeprived_AsianOther_Y7Y9_Male_Poverty | 4.84 | 0.27 | 4.31 | 5.38 |
| MostDeprived_Black_Y7Y9_otherPNS_NoPoverty | 4.82 | 0.18 | 4.46 | 5.18 |
| LeastDeprived_Mixed_Y7Y9_Male_Poverty | 4.82 | 0.38 | 4.08 | 5.56 |
| MostDeprived_AsianSA_Y10Y11_Male_Poverty | 4.81 | 0.45 | 3.94 | 5.69 |
| MidDeprived_White_Y7Y9_Male_Poverty | 4.81 | 0.43 | 3.98 | 5.65 |
| MidDeprived_AsianSA_Y10Y11_Female_NoPoverty | 4.81 | 0.30 | 4.22 | 5.40 |
| MostDeprived_AsianSA_Y10Y11_Female_NoPoverty | 4.80 | 0.29 | 4.23 | 5.37 |
| MidDeprived_Black_Y7Y9_Female_NoPoverty | 4.79 | 0.38 | 4.05 | 5.54 |
| MidDeprived_Other_Y10Y11_Male_Poverty | 4.78 | 0.35 | 4.10 | 5.47 |
| LeastDeprived_AsianOther_Y7Y9_Female_Poverty | 4.78 | 0.39 | 4.01 | 5.55 |
| LeastDeprived_Black_Y7Y9_Female_NoPoverty | 4.76 | 0.29 | 4.18 | 5.34 |
| MostDeprived_AsianSA_Y10Y11_Female_Poverty | 4.76 | 0.30 | 4.17 | 5.35 |
| MostDeprived_AsianSA_Y7Y9_Female_NoPoverty | 4.76 | 0.30 | 4.17 | 5.35 |
| MidDeprived_White_Y10Y11_otherPNS_NoPoverty | 4.74 | 0.32 | 4.11 | 5.37 |
| LeastDeprived_AsianSA_Y7Y9_Male_Poverty | 4.73 | 0.32 | 4.12 | 5.35 |
| MidDeprived_Mixed_Y7Y9_otherPNS_NoPoverty | 4.73 | 0.17 | 4.41 | 5.06 |
| MidDeprived_Black_Y7Y9_Female_Poverty | 4.73 | 0.32 | 4.11 | 5.36 |
| LeastDeprived_AsianOther_Y10Y11_Male_Poverty | 4.71 | 0.28 | 4.17 | 5.26 |
| MidDeprived_Other_Y10Y11_Female_Poverty | 4.71 | 0.19 | 4.33 | 5.09 |
| MostDeprived_Black_Y7Y9_Female_Poverty | 4.71 | 0.26 | 4.19 | 5.23 |
| MidDeprived_Mixed_Y7Y9_Male_Poverty | 4.71 | 0.30 | 4.13 | 5.29 |
| MostDeprived_Black_Y10Y11_Male_Poverty | 4.70 | 0.24 | 4.22 | 5.18 |
| MostDeprived_Other_Y7Y9_otherPNS_NoPoverty | 4.67 | 0.20 | 4.29 | 5.06 |
| LeastDeprived_AsianSA_Y7Y9_otherPNS_NoPoverty | 4.65 | 0.44 | 3.79 | 5.52 |
| MidDeprived_AsianSA_Y7Y9_otherPNS_NoPoverty | 4.63 | 0.23 | 4.18 | 5.08 |
| MidDeprived_AsianOther_Y10Y11_Female_NoPoverty | 4.63 | 0.23 | 4.17 | 5.08 |
| MostDeprived_Mixed_Y7Y9_Female_NoPoverty | 4.61 | 0.36 | 3.90 | 5.32 |
| LeastDeprived_Black_Y10Y11_Female_Poverty | 4.60 | 0.33 | 3.95 | 5.24 |
| LeastDeprived_Mixed_Y7Y9_Female_NoPoverty | 4.58 | 0.37 | 3.85 | 5.30 |
| MostDeprived_Black_Y7Y9_Male_Poverty | 4.56 | 0.36 | 3.86 | 5.26 |
| LeastDeprived_AsianSA_Y10Y11_Male_Poverty | 4.56 | 0.33 | 3.92 | 5.20 |
| MostDeprived_White_Y10Y11_Female_Poverty | 4.54 | 0.53 | 3.50 | 5.58 |
| MostDeprived_Mixed_Y10Y11_Female_NoPoverty | 4.54 | 0.29 | 3.98 | 5.10 |
| LeastDeprived_Black_Y10Y11_Male_Poverty | 4.53 | 0.27 | 4.01 | 5.05 |
| LeastDeprived_White_Y10Y11_Male_Poverty | 4.53 | 0.35 | 3.84 | 5.22 |
| MostDeprived_AsianSA_Y7Y9_otherPNS_NoPoverty | 4.52 | 0.29 | 3.96 | 5.08 |
| MidDeprived_Mixed_Y7Y9_Female_NoPoverty | 4.51 | 0.33 | 3.87 | 5.14 |
| MidDeprived_White_Y10Y11_Female_NoPoverty | 4.50 | 0.27 | 3.98 | 5.03 |
| MidDeprived_AsianOther_Y7Y9_Female_NoPoverty | 4.45 | 0.36 | 3.75 | 5.15 |
| LeastDeprived_Black_Y10Y11_Male_NoPoverty | 4.44 | 0.34 | 3.78 | 5.10 |
| MostDeprived_Mixed_Y7Y9_Male_Poverty | 4.44 | 0.27 | 3.92 | 4.96 |
| LeastDeprived_Other_Y10Y11_Male_Poverty | 4.43 | 0.32 | 3.81 | 5.05 |
| MostDeprived_AsianOther_Y10Y11_Male_NoPoverty | 4.42 | 0.26 | 3.91 | 4.93 |
| LeastDeprived_White_Y10Y11_Female_NoPoverty | 4.41 | 0.46 | 3.51 | 5.31 |
| MidDeprived_Other_Y7Y9_otherPNS_NoPoverty | 4.39 | 0.35 | 3.70 | 5.09 |
| MidDeprived_Mixed_Y10Y11_Male_Poverty | 4.39 | 0.18 | 4.03 | 4.75 |
| LeastDeprived_Black_Y10Y11_Female_NoPoverty | 4.38 | 0.27 | 3.86 | 4.91 |
| MidDeprived_Other_Y10Y11_Male_NoPoverty | 4.38 | 0.27 | 3.85 | 4.91 |
| MidDeprived_AsianOther_Y10Y11_Male_Poverty | 4.38 | 0.28 | 3.84 | 4.92 |
| MostDeprived_Black_Y10Y11_Female_NoPoverty | 4.37 | 0.36 | 3.67 | 5.07 |
| LeastDeprived_Other_Y10Y11_Female_NoPoverty | 4.35 | 0.29 | 3.78 | 4.93 |
| MostDeprived_Other_Y10Y11_Female_NoPoverty | 4.35 | 0.42 | 3.53 | 5.17 |
| MostDeprived_Other_Y7Y9_Male_Poverty | 4.33 | 0.43 | 3.49 | 5.18 |
| LeastDeprived_Other_Y7Y9_Female_NoPoverty | 4.31 | 0.31 | 3.71 | 4.92 |
| MidDeprived_AsianSA_Y7Y9_Male_Poverty | 4.31 | 0.29 | 3.74 | 4.88 |
| MostDeprived_AsianOther_Y7Y9_Male_Poverty | 4.31 | 0.20 | 3.91 | 4.70 |
| MidDeprived_Mixed_Y7Y9_Male_NoPoverty | 4.30 | 0.39 | 3.55 | 5.06 |
| MostDeprived_Mixed_Y7Y9_Male_NoPoverty | 4.28 | 0.37 | 3.55 | 5.01 |
| LeastDeprived_AsianOther_Y7Y9_Female_NoPoverty | 4.26 | 0.43 | 3.41 | 5.11 |
| LeastDeprived_AsianOther_Y7Y9_Male_NoPoverty | 4.25 | 0.31 | 3.65 | 4.86 |
| LeastDeprived_Mixed_Y10Y11_otherPNS_NoPoverty | 4.25 | 0.20 | 3.86 | 4.63 |
| MostDeprived_White_Y10Y11_Male_NoPoverty | 4.24 | 0.39 | 3.49 | 5.00 |
| MidDeprived_Mixed_Y10Y11_otherPNS_NoPoverty | 4.24 | 0.25 | 3.74 | 4.74 |
| MidDeprived_Other_Y7Y9_Male_Poverty | 4.24 | 0.19 | 3.86 | 4.61 |
| MidDeprived_Black_Y10Y11_Male_Poverty | 4.22 | 0.33 | 3.58 | 4.86 |
| MostDeprived_Other_Y7Y9_Male_NoPoverty | 4.21 | 0.32 | 3.58 | 4.85 |
| MidDeprived_AsianOther_Y10Y11_Male_NoPoverty | 4.21 | 0.26 | 3.70 | 4.73 |
| LeastDeprived_Mixed_Y10Y11_Female_NoPoverty | 4.21 | 0.30 | 3.61 | 4.80 |
| LeastDeprived_White_Y7Y9_Male_Poverty | 4.20 | 0.34 | 3.53 | 4.88 |
| MostDeprived_Other_Y7Y9_Female_NoPoverty | 4.20 | 0.35 | 3.51 | 4.89 |
| MostDeprived_Other_Y10Y11_Male_Poverty | 4.18 | 0.22 | 3.76 | 4.61 |
| MidDeprived_White_Y10Y11_Male_NoPoverty | 4.18 | 0.34 | 3.52 | 4.84 |
| MostDeprived_AsianOther_Y7Y9_Male_NoPoverty | 4.17 | 0.31 | 3.57 | 4.77 |
| MostDeprived_AsianOther_Y10Y11_Male_Poverty | 4.12 | 0.22 | 3.68 | 4.56 |
| MostDeprived_AsianSA_Y7Y9_Male_Poverty | 4.12 | 0.32 | 3.49 | 4.76 |
| MidDeprived_White_Y7Y9_Male_NoPoverty | 4.12 | 0.29 | 3.56 | 4.68 |
| MidDeprived_White_Y10Y11_Male_Poverty | 4.11 | 0.31 | 3.50 | 4.72 |
| MidDeprived_Other_Y7Y9_Female_NoPoverty | 4.10 | 0.29 | 3.53 | 4.67 |
| LeastDeprived_AsianSA_Y10Y11_Male_NoPoverty | 4.09 | 0.36 | 3.38 | 4.80 |
| MostDeprived_Mixed_Y10Y11_Male_NoPoverty | 4.07 | 0.39 | 3.30 | 4.83 |
| MostDeprived_AsianOther_Y7Y9_Female_NoPoverty | 4.06 | 0.29 | 3.49 | 4.63 |
| LeastDeprived_Mixed_Y10Y11_Male_NoPoverty | 4.05 | 0.32 | 3.43 | 4.67 |
| LeastDeprived_AsianOther_Y10Y11_Male_NoPoverty | 4.01 | 0.36 | 3.30 | 4.72 |
| MidDeprived_Black_Y10Y11_Male_NoPoverty | 4.01 | 0.25 | 3.52 | 4.49 |
| MidDeprived_Black_Y7Y9_Male_Poverty | 3.99 | 0.26 | 3.48 | 4.50 |
| MidDeprived_Black_Y10Y11_Female_NoPoverty | 3.98 | 0.20 | 3.59 | 4.37 |
| LeastDeprived_AsianOther_Y7Y9_Male_Poverty | 3.98 | 0.27 | 3.46 | 4.50 |
| MostDeprived_AsianSA_Y7Y9_Male_NoPoverty | 3.96 | 0.36 | 3.25 | 4.67 |
| MidDeprived_AsianOther_Y7Y9_Male_NoPoverty | 3.93 | 0.30 | 3.33 | 4.52 |
| MidDeprived_AsianSA_Y10Y11_Male_NoPoverty | 3.92 | 0.36 | 3.22 | 4.62 |
| LeastDeprived_AsianOther_Y10Y11_Female_NoPoverty | 3.92 | 0.26 | 3.42 | 4.43 |
| LeastDeprived_AsianSA_Y7Y9_Male_NoPoverty | 3.87 | 0.37 | 3.14 | 4.60 |
| MostDeprived_White_Y7Y9_Male_NoPoverty | 3.86 | 0.38 | 3.11 | 4.60 |
| MostDeprived_Black_Y10Y11_Male_NoPoverty | 3.84 | 0.35 | 3.16 | 4.52 |
| LeastDeprived_Other_Y7Y9_Male_NoPoverty | 3.80 | 0.45 | 2.91 | 4.68 |
| MidDeprived_Black_Y7Y9_Male_NoPoverty | 3.78 | 0.42 | 2.96 | 4.60 |
| MostDeprived_AsianSA_Y10Y11_Male_NoPoverty | 3.76 | 0.34 | 3.09 | 4.44 |
| MidDeprived_AsianSA_Y7Y9_Male_NoPoverty | 3.76 | 0.32 | 3.14 | 4.39 |
| LeastDeprived_Other_Y10Y11_Male_NoPoverty | 3.76 | 0.26 | 3.26 | 4.26 |
| MidDeprived_Mixed_Y10Y11_Male_NoPoverty | 3.73 | 0.36 | 3.03 | 4.43 |
| MidDeprived_Other_Y7Y9_Male_NoPoverty | 3.72 | 0.29 | 3.15 | 4.28 |
| LeastDeprived_Mixed_Y7Y9_Male_NoPoverty | 3.71 | 0.28 | 3.17 | 4.25 |
| MostDeprived_Other_Y10Y11_Male_NoPoverty | 3.69 | 0.35 | 3.01 | 4.37 |
| MostDeprived_White_Y7Y9_Male_Poverty | 3.58 | 0.51 | 2.57 | 4.59 |
| LeastDeprived_Black_Y7Y9_Male_NoPoverty | 3.47 | 0.43 | 2.63 | 4.32 |
| LeastDeprived_White_Y7Y9_Male_NoPoverty | 3.45 | 0.32 | 2.82 | 4.08 |
| LeastDeprived_White_Y10Y11_Male_NoPoverty | 3.42 | 0.26 | 2.91 | 3.92 |
| MostDeprived_Black_Y7Y9_Male_NoPoverty | 3.20 | 0.30 | 2.60 | 3.80 |

*Note*. Emotional problems indexed by the Revised Child Depression and Anxiety Scale (RCADS-11). 95% approximate CI. Stratum label order: school-level deprivation, ethnicity, school year group, gender, household poverty. AsianSA = South Asian; Other = other ethnicity groups; otherPNS = other and prefer not to say. Estimates derived from Model 3.

**Supplementary Table 5.** Predicted stratum-level deviations (**μ*_1j_***) from the average maltreatment effect

| **Cluster** | **μ*_1j_*** | ***SE*** | **CI Low** | **CI High** |
| --- | --- | --- | --- | --- |
| MostDeprived_Black_Y7Y9_Male_NoPoverty | -1.40 | 0.34 | -2.08 | -0.73 |
| LeastDeprived_White_Y7Y9_Male_NoPoverty | -1.27 | 0.21 | -1.68 | -0.86 |
| LeastDeprived_White_Y10Y11_Male_NoPoverty | -1.22 | 0.35 | -1.91 | -0.54 |
| LeastDeprived_Black_Y7Y9_Male_NoPoverty | -1.20 | 0.38 | -1.93 | -0.46 |
| MostDeprived_White_Y7Y9_Male_Poverty | -1.08 | 0.37 | -1.81 | -0.35 |
| MostDeprived_Other_Y10Y11_Male_NoPoverty | -1.06 | 0.35 | -1.75 | -0.37 |
| MidDeprived_Mixed_Y10Y11_Male_NoPoverty | -1.02 | 0.25 | -1.50 | -0.54 |
| LeastDeprived_Mixed_Y7Y9_Male_NoPoverty | -1.02 | 0.21 | -1.42 | -0.61 |
| LeastDeprived_Other_Y10Y11_Male_NoPoverty | -1.02 | 0.33 | -1.65 | -0.38 |
| MidDeprived_Other_Y7Y9_Male_NoPoverty | -0.99 | 0.33 | -1.63 | -0.36 |
| MidDeprived_AsianSA_Y7Y9_Male_NoPoverty | -0.98 | 0.37 | -1.70 | -0.26 |
| MostDeprived_AsianSA_Y10Y11_Male_NoPoverty | -0.93 | 0.24 | -1.40 | -0.47 |
| LeastDeprived_Other_Y7Y9_Male_NoPoverty | -0.90 | 0.34 | -1.56 | -0.24 |
| MostDeprived_White_Y7Y9_Male_NoPoverty | -0.89 | 0.27 | -1.41 | -0.36 |
| LeastDeprived_AsianSA_Y7Y9_Male_NoPoverty | -0.80 | 0.40 | -1.59 | -0.00 |
| MidDeprived_Black_Y7Y9_Male_NoPoverty | -0.79 | 0.33 | -1.45 | -0.14 |
| MidDeprived_AsianOther_Y7Y9_Male_NoPoverty | -0.79 | 0.28 | -1.34 | -0.24 |
| LeastDeprived_AsianOther_Y10Y11_Female_NoPoverty | -0.76 | 0.27 | -1.28 | -0.24 |
| MostDeprived_AsianSA_Y7Y9_Male_NoPoverty | -0.76 | 0.32 | -1.38 | -0.13 |
| MostDeprived_Black_Y10Y11_Male_NoPoverty | -0.75 | 0.34 | -1.41 | -0.09 |
| MidDeprived_Black_Y10Y11_Female_NoPoverty | -0.72 | 0.21 | -1.14 | -0.30 |
| MidDeprived_Black_Y7Y9_Male_Poverty | -0.71 | 0.29 | -1.28 | -0.14 |
| LeastDeprived_AsianOther_Y10Y11_Male_NoPoverty | -0.69 | 0.27 | -1.21 | -0.17 |
| MidDeprived_Black_Y10Y11_Male_NoPoverty | -0.69 | 0.31 | -1.29 | -0.09 |
| LeastDeprived_Mixed_Y10Y11_Male_NoPoverty | -0.68 | 0.28 | -1.23 | -0.14 |
| LeastDeprived_AsianOther_Y7Y9_Male_Poverty | -0.68 | 0.29 | -1.24 | -0.11 |
| MostDeprived_AsianOther_Y7Y9_Female_NoPoverty | -0.67 | 0.23 | -1.12 | -0.23 |
| MidDeprived_AsianSA_Y10Y11_Male_NoPoverty | -0.67 | 0.26 | -1.18 | -0.16 |
| LeastDeprived_AsianSA_Y10Y11_Male_NoPoverty | -0.65 | 0.24 | -1.13 | -0.18 |
| MostDeprived_White_Y10Y11_Male_NoPoverty | -0.61 | 0.37 | -1.33 | 0.11 |
| MostDeprived_AsianOther_Y10Y11_Male_Poverty | -0.59 | 0.13 | -0.86 | -0.33 |
| MidDeprived_White_Y10Y11_Male_Poverty | -0.58 | 0.27 | -1.11 | -0.05 |
| MostDeprived_Mixed_Y10Y11_Male_NoPoverty | -0.57 | 0.36 | -1.27 | 0.14 |
| MidDeprived_White_Y7Y9_Male_NoPoverty | -0.56 | 0.25 | -1.05 | -0.07 |
| MostDeprived_AsianOther_Y7Y9_Male_NoPoverty | -0.56 | 0.22 | -0.99 | -0.13 |
| MidDeprived_Other_Y7Y9_Female_NoPoverty | -0.55 | 0.15 | -0.84 | -0.27 |
| MostDeprived_Other_Y7Y9_Male_NoPoverty | -0.54 | 0.33 | -1.20 | 0.11 |
| MostDeprived_Other_Y7Y9_Female_NoPoverty | -0.53 | 0.41 | -1.34 | 0.28 |
| MostDeprived_AsianSA_Y7Y9_Male_Poverty | -0.49 | 0.24 | -0.96 | -0.03 |
| MidDeprived_AsianOther_Y10Y11_Male_NoPoverty | -0.49 | 0.26 | -1.00 | 0.03 |
| MostDeprived_Mixed_Y7Y9_Male_NoPoverty | -0.49 | 0.24 | -0.95 | -0.03 |
| MostDeprived_Other_Y10Y11_Male_Poverty | -0.48 | 0.20 | -0.88 | -0.09 |
| MidDeprived_Other_Y7Y9_Male_Poverty | -0.48 | 0.15 | -0.78 | -0.19 |
| MidDeprived_Black_Y10Y11_Male_Poverty | -0.47 | 0.35 | -1.16 | 0.22 |
| LeastDeprived_Mixed_Y10Y11_Female_NoPoverty | -0.47 | 0.17 | -0.81 | -0.13 |
| LeastDeprived_AsianOther_Y7Y9_Male_NoPoverty | -0.46 | 0.32 | -1.09 | 0.18 |
| MidDeprived_Mixed_Y10Y11_otherPNS_NoPoverty | -0.45 | 0.27 | -0.98 | 0.07 |
| MidDeprived_White_Y10Y11_Male_NoPoverty | -0.45 | 0.37 | -1.18 | 0.28 |
| MidDeprived_Mixed_Y7Y9_Male_NoPoverty | -0.44 | 0.29 | -1.01 | 0.12 |
| LeastDeprived_Mixed_Y10Y11_otherPNS_NoPoverty | -0.44 | 0.24 | -0.90 | 0.03 |
| LeastDeprived_AsianOther_Y7Y9_Female_NoPoverty | -0.42 | 0.26 | -0.93 | 0.08 |
| LeastDeprived_White_Y7Y9_Male_Poverty | -0.40 | 0.36 | -1.11 | 0.30 |
| LeastDeprived_White_Y10Y11_Female_NoPoverty | -0.36 | 0.41 | -1.16 | 0.44 |
| MidDeprived_AsianOther_Y7Y9_Female_NoPoverty | -0.35 | 0.25 | -0.85 | 0.14 |
| LeastDeprived_Other_Y7Y9_Female_NoPoverty | -0.35 | 0.26 | -0.86 | 0.16 |
| MostDeprived_Other_Y7Y9_Male_Poverty | -0.35 | 0.30 | -0.93 | 0.23 |
| MidDeprived_Other_Y7Y9_otherPNS_NoPoverty | -0.34 | 0.30 | -0.92 | 0.24 |
| MostDeprived_AsianOther_Y7Y9_Male_Poverty | -0.34 | 0.16 | -0.65 | -0.03 |
| MidDeprived_Mixed_Y10Y11_Male_Poverty | -0.32 | 0.18 | -0.67 | 0.02 |
| MostDeprived_AsianOther_Y10Y11_Male_NoPoverty | -0.32 | 0.24 | -0.79 | 0.16 |
| MidDeprived_AsianSA_Y7Y9_Male_Poverty | -0.31 | 0.29 | -0.89 | 0.26 |
| MostDeprived_Black_Y10Y11_Female_NoPoverty | -0.30 | 0.18 | -0.67 | 0.06 |
| MostDeprived_Other_Y10Y11_Female_NoPoverty | -0.30 | 0.28 | -0.85 | 0.25 |
| MidDeprived_Other_Y10Y11_Male_NoPoverty | -0.29 | 0.26 | -0.80 | 0.23 |
| LeastDeprived_Black_Y10Y11_Female_NoPoverty | -0.28 | 0.26 | -0.78 | 0.22 |
| MostDeprived_Mixed_Y7Y9_Male_Poverty | -0.27 | 0.16 | -0.58 | 0.04 |
| MidDeprived_Mixed_Y7Y9_Female_NoPoverty | -0.26 | 0.22 | -0.69 | 0.16 |
| LeastDeprived_Other_Y10Y11_Female_NoPoverty | -0.26 | 0.19 | -0.63 | 0.12 |
| MidDeprived_White_Y10Y11_Female_NoPoverty | -0.24 | 0.26 | -0.74 | 0.27 |
| LeastDeprived_Black_Y10Y11_Male_NoPoverty | -0.22 | 0.44 | -1.08 | 0.63 |
| MidDeprived_AsianOther_Y10Y11_Male_Poverty | -0.22 | 0.27 | -0.74 | 0.31 |
| MostDeprived_Black_Y7Y9_Male_Poverty | -0.20 | 0.31 | -0.80 | 0.41 |
| LeastDeprived_Mixed_Y7Y9_Female_NoPoverty | -0.18 | 0.21 | -0.60 | 0.23 |
| LeastDeprived_Other_Y10Y11_Male_Poverty | -0.18 | 0.39 | -0.94 | 0.58 |
| MostDeprived_Mixed_Y10Y11_Female_NoPoverty | -0.18 | 0.26 | -0.69 | 0.34 |
| MostDeprived_White_Y10Y11_Female_Poverty | -0.16 | 0.31 | -0.78 | 0.45 |
| LeastDeprived_White_Y10Y11_Male_Poverty | -0.12 | 0.37 | -0.85 | 0.61 |
| LeastDeprived_AsianSA_Y10Y11_Male_Poverty | -0.11 | 0.27 | -0.64 | 0.42 |
| MostDeprived_AsianSA_Y7Y9_otherPNS_NoPoverty | -0.09 | 0.27 | -0.62 | 0.44 |
| LeastDeprived_AsianSA_Y7Y9_otherPNS_NoPoverty | -0.08 | 0.21 | -0.50 | 0.33 |
| MidDeprived_AsianOther_Y10Y11_Female_NoPoverty | -0.06 | 0.11 | -0.29 | 0.16 |
| LeastDeprived_AsianSA_Y7Y9_Male_Poverty | -0.04 | 0.18 | -0.39 | 0.32 |
| MidDeprived_Mixed_Y7Y9_Male_Poverty | -0.03 | 0.37 | -0.75 | 0.69 |
| MostDeprived_Black_Y10Y11_Male_Poverty | -0.03 | 0.26 | -0.54 | 0.47 |
| LeastDeprived_Black_Y10Y11_Male_Poverty | -0.03 | 0.28 | -0.58 | 0.53 |
| MostDeprived_Black_Y7Y9_Female_Poverty | -0.03 | 0.28 | -0.57 | 0.52 |
| LeastDeprived_Black_Y10Y11_Female_Poverty | -0.02 | 0.22 | -0.45 | 0.42 |
| LeastDeprived_AsianOther_Y10Y11_Male_Poverty | -0.00 | 0.14 | -0.29 | 0.28 |
| MostDeprived_Other_Y7Y9_otherPNS_NoPoverty | 0.01 | 0.07 | -0.13 | 0.14 |
| MidDeprived_AsianSA_Y7Y9_otherPNS_NoPoverty | 0.02 | 0.19 | -0.36 | 0.40 |
| MostDeprived_Mixed_Y7Y9_Female_NoPoverty | 0.02 | 0.39 | -0.74 | 0.78 |
| MidDeprived_AsianOther_Y7Y9_Male_Poverty | 0.02 | 0.27 | -0.51 | 0.55 |
| MidDeprived_Other_Y10Y11_Female_Poverty | 0.02 | 0.04 | -0.06 | 0.10 |
| LeastDeprived_Black_Y7Y9_Female_NoPoverty | 0.07 | 0.34 | -0.60 | 0.74 |
| MidDeprived_Mixed_Y7Y9_otherPNS_NoPoverty | 0.07 | 0.09 | -0.10 | 0.24 |
| LeastDeprived_AsianOther_Y7Y9_Female_Poverty | 0.07 | 0.30 | -0.51 | 0.65 |
| MostDeprived_AsianSA_Y7Y9_Female_NoPoverty | 0.08 | 0.27 | -0.45 | 0.60 |
| MidDeprived_Black_Y7Y9_Female_Poverty | 0.08 | 0.18 | -0.28 | 0.44 |
| MidDeprived_Other_Y10Y11_Male_Poverty | 0.09 | 0.29 | -0.47 | 0.65 |
| MostDeprived_AsianSA_Y10Y11_Female_Poverty | 0.09 | 0.17 | -0.25 | 0.43 |
| MostDeprived_Black_Y7Y9_otherPNS_NoPoverty | 0.10 | 0.08 | -0.05 | 0.25 |
| MostDeprived_AsianSA_Y10Y11_Male_Poverty | 0.11 | 0.22 | -0.33 | 0.55 |
| MidDeprived_White_Y10Y11_otherPNS_NoPoverty | 0.12 | 0.22 | -0.30 | 0.54 |
| MidDeprived_Black_Y7Y9_Female_NoPoverty | 0.16 | 0.38 | -0.59 | 0.90 |
| MidDeprived_White_Y7Y9_Male_Poverty | 0.17 | 0.30 | -0.42 | 0.76 |
| LeastDeprived_Other_Y7Y9_Female_Poverty | 0.20 | 0.27 | -0.33 | 0.73 |
| MostDeprived_AsianSA_Y10Y11_Female_NoPoverty | 0.20 | 0.38 | -0.54 | 0.94 |
| LeastDeprived_Mixed_Y7Y9_Male_Poverty | 0.21 | 0.31 | -0.41 | 0.83 |
| MidDeprived_AsianSA_Y7Y9_Female_NoPoverty | 0.22 | 0.27 | -0.31 | 0.75 |
| MidDeprived_AsianSA_Y10Y11_otherPNS_NoPoverty | 0.22 | 0.13 | -0.04 | 0.48 |
| LeastDeprived_AsianSA_Y10Y11_Female_Poverty | 0.23 | 0.21 | -0.17 | 0.64 |
| MidDeprived_AsianSA_Y10Y11_Male_Poverty | 0.25 | 0.20 | -0.14 | 0.65 |
| LeastDeprived_AsianSA_Y10Y11_Female_NoPoverty | 0.26 | 0.25 | -0.23 | 0.75 |
| LeastDeprived_Black_Y7Y9_Male_Poverty | 0.26 | 0.15 | -0.03 | 0.55 |
| MidDeprived_White_Y7Y9_Female_NoPoverty | 0.26 | 0.34 | -0.41 | 0.93 |
| MidDeprived_AsianSA_Y7Y9_Female_Poverty | 0.28 | 0.35 | -0.40 | 0.96 |
| MostDeprived_AsianOther_Y7Y9_otherPNS_Poverty | 0.28 | 0.10 | 0.09 | 0.48 |
| MidDeprived_AsianSA_Y10Y11_Female_NoPoverty | 0.29 | 0.19 | -0.09 | 0.66 |
| LeastDeprived_Mixed_Y7Y9_Female_Poverty | 0.29 | 0.19 | -0.09 | 0.67 |
| MostDeprived_Mixed_Y7Y9_otherPNS_NoPoverty | 0.29 | 0.28 | -0.25 | 0.83 |
| MostDeprived_White_Y10Y11_Female_NoPoverty | 0.30 | 0.33 | -0.36 | 0.96 |
| MidDeprived_Other_Y7Y9_Female_Poverty | 0.32 | 0.24 | -0.15 | 0.78 |
| MidDeprived_Other_Y10Y11_Female_NoPoverty | 0.32 | 0.18 | -0.04 | 0.68 |
| MidDeprived_White_Y10Y11_Female_Poverty | 0.32 | 0.34 | -0.34 | 0.98 |
| MostDeprived_AsianOther_Y7Y9_otherPNS_NoPoverty | 0.35 | 0.21 | -0.06 | 0.75 |
| MostDeprived_Black_Y7Y9_Female_NoPoverty | 0.38 | 0.43 | -0.48 | 1.23 |
| LeastDeprived_White_Y10Y11_Female_Poverty | 0.38 | 0.43 | -0.47 | 1.23 |
| MostDeprived_Mixed_Y10Y11_Male_Poverty | 0.41 | 0.20 | 0.02 | 0.79 |
| MostDeprived_AsianOther_Y10Y11_Female_Poverty | 0.42 | 0.26 | -0.09 | 0.92 |
| MidDeprived_AsianOther_Y10Y11_Female_Poverty | 0.42 | 0.14 | 0.15 | 0.68 |
| MostDeprived_White_Y7Y9_otherPNS_NoPoverty | 0.44 | 0.30 | -0.15 | 1.04 |
| MidDeprived_Mixed_Y10Y11_Female_Poverty | 0.45 | 0.14 | 0.18 | 0.72 |
| MostDeprived_Black_Y10Y11_Female_Poverty | 0.46 | 0.23 | 0.02 | 0.90 |
| MostDeprived_Mixed_Y7Y9_Female_Poverty | 0.46 | 0.34 | -0.21 | 1.14 |
| LeastDeprived_Other_Y10Y11_Female_Poverty | 0.46 | 0.35 | -0.21 | 1.14 |
| MidDeprived_AsianOther_Y7Y9_Female_Poverty | 0.47 | 0.09 | 0.28 | 0.65 |
| MostDeprived_Mixed_Y10Y11_Female_Poverty | 0.48 | 0.21 | 0.06 | 0.89 |
| LeastDeprived_Mixed_Y7Y9_otherPNS_Poverty | 0.49 | 0.17 | 0.16 | 0.82 |
| LeastDeprived_AsianSA_Y7Y9_Female_Poverty | 0.51 | 0.18 | 0.15 | 0.86 |
| MostDeprived_White_Y10Y11_otherPNS_NoPoverty | 0.51 | 0.23 | 0.05 | 0.96 |
| MostDeprived_AsianOther_Y10Y11_Female_NoPoverty | 0.51 | 0.21 | 0.10 | 0.91 |
| MidDeprived_Black_Y10Y11_Female_Poverty | 0.52 | 0.17 | 0.20 | 0.85 |
| MidDeprived_AsianSA_Y10Y11_Female_Poverty | 0.54 | 0.25 | 0.05 | 1.02 |
| MostDeprived_Other_Y10Y11_Female_Poverty | 0.54 | 0.25 | 0.06 | 1.03 |
| LeastDeprived_Other_Y7Y9_Male_Poverty | 0.55 | 0.31 | -0.07 | 1.16 |
| LeastDeprived_AsianOther_Y10Y11_Female_Poverty | 0.56 | 0.22 | 0.12 | 0.99 |
| LeastDeprived_AsianSA_Y10Y11_otherPNS_NoPoverty | 0.57 | 0.22 | 0.14 | 0.99 |
| MidDeprived_Mixed_Y7Y9_otherPNS_Poverty | 0.57 | 0.19 | 0.20 | 0.95 |
| LeastDeprived_Mixed_Y10Y11_Female_Poverty | 0.59 | 0.23 | 0.14 | 1.03 |
| LeastDeprived_White_Y7Y9_Female_Poverty | 0.59 | 0.29 | 0.02 | 1.15 |
| LeastDeprived_AsianOther_Y7Y9_otherPNS_NoPoverty | 0.59 | 0.14 | 0.31 | 0.87 |
| MidDeprived_Mixed_Y10Y11_Female_NoPoverty | 0.59 | 0.13 | 0.35 | 0.84 |
| MostDeprived_AsianOther_Y7Y9_Female_Poverty | 0.61 | 0.28 | 0.07 | 1.15 |
| MidDeprived_White_Y7Y9_Female_Poverty | 0.63 | 0.36 | -0.08 | 1.33 |
| MostDeprived_White_Y10Y11_Male_Poverty | 0.66 | 0.33 | 0.01 | 1.31 |
| LeastDeprived_Mixed_Y10Y11_Male_Poverty | 0.68 | 0.28 | 0.14 | 1.22 |
| LeastDeprived_AsianSA_Y10Y11_otherPNS_Poverty | 0.69 | 0.29 | 0.13 | 1.25 |
| LeastDeprived_Mixed_Y7Y9_otherPNS_NoPoverty | 0.70 | 0.15 | 0.40 | 0.99 |
| LeastDeprived_White_Y7Y9_otherPNS_NoPoverty | 0.71 | 0.24 | 0.24 | 1.18 |
| MostDeprived_Other_Y7Y9_Female_Poverty | 0.71 | 0.34 | 0.05 | 1.37 |
| MostDeprived_Other_Y7Y9_otherPNS_Poverty | 0.72 | 0.16 | 0.40 | 1.04 |
| LeastDeprived_Black_Y7Y9_Female_Poverty | 0.74 | 0.28 | 0.20 | 1.29 |
| MidDeprived_White_Y7Y9_otherPNS_Poverty | 0.76 | 0.40 | -0.03 | 1.54 |
| LeastDeprived_Mixed_Y10Y11_otherPNS_Poverty | 0.76 | 0.16 | 0.44 | 1.08 |
| LeastDeprived_White_Y7Y9_otherPNS_Poverty | 0.77 | 0.29 | 0.20 | 1.34 |
| LeastDeprived_White_Y10Y11_otherPNS_NoPoverty | 0.82 | 0.32 | 0.19 | 1.45 |
| LeastDeprived_AsianOther_Y10Y11_otherPNS_NoPoverty | 0.82 | 0.20 | 0.43 | 1.21 |
| LeastDeprived_AsianSA_Y7Y9_Female_NoPoverty | 0.84 | 0.35 | 0.15 | 1.54 |
| MidDeprived_Mixed_Y7Y9_Female_Poverty | 0.85 | 0.28 | 0.29 | 1.40 |
| MostDeprived_White_Y7Y9_otherPNS_Poverty | 0.86 | 0.30 | 0.27 | 1.44 |
| MidDeprived_White_Y7Y9_otherPNS_NoPoverty | 0.87 | 0.28 | 0.32 | 1.42 |
| MostDeprived_White_Y7Y9_Female_Poverty | 0.88 | 0.39 | 0.11 | 1.64 |
| MidDeprived_White_Y10Y11_otherPNS_Poverty | 0.89 | 0.22 | 0.46 | 1.32 |
| MostDeprived_White_Y7Y9_Female_NoPoverty | 0.93 | 0.27 | 0.41 | 1.46 |
| MostDeprived_Black_Y7Y9_otherPNS_Poverty | 1.03 | 0.25 | 0.54 | 1.53 |
| MostDeprived_Mixed_Y7Y9_otherPNS_Poverty | 1.15 | 0.33 | 0.51 | 1.80 |
| LeastDeprived_White_Y10Y11_otherPNS_Poverty | 1.23 | 0.44 | 0.37 | 2.09 |
| MostDeprived_White_Y10Y11_otherPNS_Poverty | 1.28 | 0.39 | 0.52 | 2.04 |
| MostDeprived_AsianSA_Y7Y9_Female_Poverty | 1.39 | 0.26 | 0.87 | 1.90 |
| LeastDeprived_White_Y7Y9_Female_NoPoverty | 1.51 | 0.28 | 0.97 | 2.06 |

*Note*. Emotional problems indexed by the Revised Child Depression and Anxiety Scale (RCADS-11). 95% approximate CI. Stratum label order: school-level deprivation, ethnicity, school year group, gender, household poverty. AsianSA = South Asian; Other = other ethnicity groups; otherPNS = other and prefer not to say. Estimates derived from Model 3.
